# Exploiting Co-Benefits of Increased Rice Production and Reduced Greenhouse Gas Emission through Optimized Crop and Soil Management

**DOI:** 10.1371/journal.pone.0140023

**Published:** 2015-10-09

**Authors:** Ning An, Mingsheng Fan, Fusuo Zhang, Peter Christie, Jianchang Yang, Jianliang Huang, Shiwei Guo, Xiaojun Shi, Qiyuan Tang, Jianwei Peng, Xuhua Zhong, Yixiang Sun, Shihua Lv, Rongfeng Jiang, Achim Dobermann

**Affiliations:** 1 Centre for Resources, Environment and Food Security, College of Resources and Environmental Sciences, China Agricultural University, Beijing, China; 2 Key Laboratory of Crop Genetics and Physiology of Jiangsu Province, Yangzhou University, Yangzhou, China; 3 College of Plant Science & Technology, Huazhong Agricultural University, Wuhan, China; 4 College of Resources & Environmental Sciences, Nanjing Agricultural University, Nanjing, China; 5 Resource and Environment College, Southwest University, Chongqing, China; 6 Crop Physiology, Ecology & Production Center, Hunan Agricultural University, Changsha, China; 7 Rice Research Institute, Guangdong Academy of Agricultural Sciences, Guangzhou, Guangdong, China; 8 Soil &Fertilizer Research Institute, Anhui Academy of Agricultural Sciences, Hefei, China; 9 Institute of Soils and Fertilizers, Sichuan Academy of Agricultural Sciences, Chengdu, China; 10 Rothamsted Research, Harpenden, Herts, United Kingdom; Chinese Academy of Sciences, CHINA

## Abstract

Meeting the future food security challenge without further sacrificing environmental integrity requires transformative changes in managing the key biophysical determinants of increasing agronomic productivity and reducing the environmental footprint. Here, we focus on Chinese rice production and quantitatively address this concern by conducting 403 on-farm trials across diverse rice farming systems. Inherent soil productivity, management practices and rice farming type resulted in confounded and interactive effects on yield, yield gaps and greenhouse gas (GHG) emissions (N_2_O, CH_4_ and CO_2_-equivalent) with both trade-offs and compensating effects. Advances in nitrogen, water and crop management (Best Management Practices—BMPs) helped closing existing yield gaps and resulted in a substantial reduction in CO_2_-equivalent emission of rice farming despite a tradeoff of increase N_2_O emission. However, inherent soil properties limited rice yields to a larger extent than previously known. Cultivating inherently better soil also led to lower GHG intensity (GHG emissions per unit yield). Neither adopting BMPs only nor improving soils with low or moderate productivity alone can adequately address the challenge of substantially increasing rice production while reducing the environmental footprint. A combination of both represents the most efficient strategy to harness the combined-benefits of enhanced production and mitigating climate change. Extrapolating from our farm data, this strategy could increase rice production in China by 18%, which would meet the demand for direct human consumption of rice by 2030. It would also reduce fertilizer nitrogen consumption by 22% and decrease CO_2_-equivalent emissions during the rice growing period by 7% compared with current farming practice continues. Benefits vary by rice-based cropping systems. Single rice systems have the largest food provision benefits due to its wider yield gap and total cultivated area, whereas double-rice system (especially late rice) contributes primarily to reducing GHG emissions. The study therefore provides farm-based evidence for feasible, practical approaches towards achieving realistic food security and environmental quality targets at a national scale.

## Introduction

Global aggregate food production needs to increase by at least 60–70% by 2050 to meet the projected food demands from population growth and economic development [1]. Actual crop production targets vary widely by countries, but it is generally acknowledged that the increase in production must largely come from higher yields on currently cultivated land to avoid further environmental degradation, destruction of natural ecosystems and loss of biodiversity [1,2].

Rice (*Oryza sativa* L.) is the most important food crop in the developing world and is the staple food of more than half of the global population, many of whom are also extremely vulnerable to high rice prices [3]. Future global food security and the precarious livelihoods of the world’s poor will no doubt depend on maintaining reliable growth in rice productivity and production. However, rice farming systems are facing unprecedented challenges and risks. Recent studies show that both average yield stagnation and large yield gaps (e.g. 2000–5000 kg ha^-1^) often occur together across and within major rice production regions [4–10]. Breaking the yield barriers is therefore a major challenge.

Even bigger challenges in rice farming are whether or to what extent the future growth in rice production can be decoupled from inefficient and unsustainable use of primary resources—especially nitrogen (N) and water—and consequently reduce environmental footprints. The problem may be especially serious in China, a country which accounts for about 19% of the global area under rice cultivation and 29% of global rice production but uses about 36% of the total fertilizer N used for rice production worldwide [11,12]. Shortage of irrigation water will be a main concern for future rice cropping systems and this is especially serious in China and requires rethinking of the current management paradigms [13,14]. However, water saving technology for rice production offers opportunities to reduce emissions of CH_4_, a major greenhouse gas in paddy soils, but carries a risk of higher N_2_O emissions [15,16]. The global warming potential (GWP, the sum of CH_4_ and N_2_O emissions expressed as CO_2_ equivalents, CO_2_-eq) and greenhouse gas intensity (GHGI, CO_2_-eq per unit yield) of such management changes would be highly uncertain. They depend on agricultural management factors such as fertilizer N application rate and specific irrigation management practices [15,17,18] as well as environmental factors such as soil pH and soil organic carbon content (SOC) [19]. GHGI also could be affected by rice yield [20].

Field studies show the potential for achieving high rice yields in combination with high N use efficiencies and low environmental impacts by adopting good crop and nutrient management practices [10,21–24]. However, most of these studies have focused on crop management practices and have not adequately addressed biophysical constraints associated with the soil resource base as a key determinant of productivity and environmental impact. Many field experiments have been conducted at research stations or in selected farmers’ fields which often situated in areas of fertile soils with favorable topography, which raises concerns about the broader applicability of the results obtained.

On the other hand, global or regional scale studies using models also fail to integrate soils into the analysis because of unavailability and poor quality of soil data and difficulties in linking specific (or a set of) soil properties to crop yields [7,25]. Various forms of land degradation often coincide with areas of extreme poverty [26] and the perspective of meeting the growing demand for rice may be more optimistic than the available soils could support.

Further, rice is practiced over a wide range of agroecological zones with very different climate conditions and growing environment [27]. It is unclear what the realistic of optimum crop and N management with less water might be across diverse rice farming systems. There is an urgent need to acquire a fundamental understanding of the potential significance of biophysical factors versus management factors in achieving the multiple goals of increasing rice productivity and production, and restoring environmental integrity, at a scale that enables land managers to use this information for concrete action in farmers’ fields. Comparing various management treatments in a series of farmers’ fields may represent the first step and most conceptually straightforward way toward this goal.

With a focus on China, the main objectives of the present study were to quantify and understand the interactive effects of biophysical factors (e.g. soil and rice farming type) and crop management practices on agronomic productivity and environmental impacts, and to evaluate total rice production, fertilizer N consumption and emissions of two major greenhouse gases (GHGs, N_2_O and CH_4_) for major rice farming types and at national scale following alternative strategies. In that context crop management practices are referred to as current farming practice (FP) and best management practice (BMP), with the latter representing relatively low-cost easily adoptable practices such as improved N and water management, cultivating healthy seedlings and increasing rice transplanting density. Our central hypothesis was that addressing adequately the challenge of closing existing rice yield gap and increase rice production while reducing the environmental footprint will depend on the exploitation of the synergistic benefits of advances in nutrient, water and crop management practices and enhanced inherent soil productivity.

## Materials and Methods

### Cropping systems

The major rice systems in the current study were double-cropping of early and late rice in south China (18–26°N, 110–116°E, warm/cool humid subtropics) and single rice cropping in the Yangtze River Basin (30–31°N,117–121°E, warm sub-humid subtropics) [27]. Early and late rice are both grown in the same field each year (early rice from early April to July and late rice from July to late October), whereas single crop rice in the Yangtze Delta is grown from late May to late September and rotated with other upland crops. These systems account for about 82% of Chinese rice production [28].

### Data description

#### On-farm trials

On-farm trials (n = 403), were conducted with three treatments, namely FP, BMP and zero-N (to estimate inherent soil productivity) in the major rice production provinces Hunan (n = 205), Hubei (n = 32), Guangdong (n = 28), Anhui (n = 44), Jiangsu (n = 59) and Chongqing (n = 35) from 2008 to 2011. No specific permissions were required for doing these on-farm trials in each location, because all locations are located in major Chinese rice production domains. The field studies did not involve endangered or protected species. These on-farm trials were conducted for 1–2 years on selected soils representing different inherent productivities. The geographical distribution of the sites is shown in supporting materials (S1 Fig). Of the 403 trials, 246 were double rice and 157 single rice systems. Average farm size ranged from to 0.2 ha to 0.4 ha. In FP the farmers applied all management practices based on their own decisions. The BMPs were designed to represent feasible and practical measures which could be adopted widely in the near and medium future in Chinese rice farming. BMPs included: (1) improving nutrient management based on the principles of integrated, site-specific nutrient management [23,29,30], (2) managing water by an intermittent irrigation pattern (F-D-F-M) characterized by flooding—midseason drainage—re-flooding—alternate drying-wetting during the subsequent period [31], and (3) increasing transplanting density to ensure high-yielding rice populations. All crop management practices with exception of N fertilization in zero-N plots were same as BMPs. The details of FPs and BMPs are documented in supporting materials (Table A in S1 Text).

Plant sampling followed standard procedures at all locations and grain yields were reported at a grain moisture content of 14% [32].

#### Literature survey

We estimated the emissions of N_2_O and CH_4_ during the rice growing season based on the above on-farm trails. To establish empirical N_2_O emission models for both FP (traditional flooding water regimes, F-D-F) and BMP (F-D-F-M) systems, the literature survey focused on field measurements of N_2_O emissions in relation to N fertilizer application rates of double rice in south China and single rice in the Yangtze Delta. The details of the data and the references involved in the analysis are showed in supporting materials (Table B in S1 Text). Of the 95 observations in 35 fields in 29 published sources, 51 represented F-D-F and 44 F-D-F-M. These represented as complete and as large a database of N_2_O emissions to N rate for F-D-F and F-D-F-M as was possible for the major Chinese paddy soils. CH_4_ emissions were estimated based on previous developing statistical model [19].

The assessment of the current status of inherent productivity of paddy soils at national scale depended on yield data during 1–2 years in zero-N plots derived from 5351 trials conducted from 2000 to 2010 across the double rice region in the south and single rice systems in the Yangtze Delta. A total of 177 published papers and documents from which the data were derived are listed in supporting materials (Table C in S1 Text). The geographical distribution of the sites is shown in supporting materials (S1 Fig).

### Data manipulation

We carried out the following steps utilizing on-farm trials and literature-based datasets.

1. Classification of inherent soil productivity of major rice production systems by plant-based approach [33].
2. Establishment of statistical models of N_2_O emissions for both FP and BMP. Based on existing literature-based datasets linear and exponential models were used to evaluate the relationship between N_2_O emissions and N rate. Regression efficiencies (corrected R^2^) were used to identify the best fit curve. On the basis of the N_2_O loss response curves, N_2_O emission was calculated for both FP and BMP for each of 403 on-farm trails. The CH_4_ emission was calculated based on soil inherent productivity grades at rice farming type scale by using existing model [19].
3. Checking the potential importance and interactive effects of soil inherent productivity, management practices (FP vs BMP) and rice farming type on yield, yield gap, N_2_O and CH_4_ emissions, GWP and GHGI based on 403 on-farm trials. In our study we defined yield gap as the difference between yield under FP (on soils with various inherent productivity) and the attainable yield, defined as the mean yield of the 20% highest-yielding locations under BMPs. The GWP and GHGI are the sum of N_2_O and CH_4_ emissions over a 100-year time horizon at the area and yield scale from rice fields during the rice growing season.
4. Calculating the responses of BMPs upon adoption on soils of different inherent productivity levels for three major rice farming systems. The response of BMPs is defined as the difference in agronomic and economic performance and environmental impact between BMPs and FPs.
5. Evaluating total rice production, fertilizer N consumption, and greenhouse gas emissions for the three major cropping systems by synthesizing the areas of various soils of inherent productivity and agronomic performances and environmental impacts of management practices on corresponding soils following four strategies (continuing current FPs, adoption of BMPs, improving soils of low and moderate productivity, and a combination of improving soils of low and moderate productivity and adoption of BMPs).

### Data statistics

Means of management treatments for grain yield, N application, and GHG emission and regression slopes of yield, yield gap and GHG emission to inherent soil productivity were compared at a 0.05 level of significance at rice farming type scale. All statistical analysis was performed using the SPSS software package (SPSS 13.0, SPSS Inc).

Detailed descriptions of the data treatments and analysis are given in the S1 Text as Supplementary materials and methods.

## Results

### Interactive effects of soil inherent productivity, rice farming type and management practices on yield and yield gap

**Fig 1:**
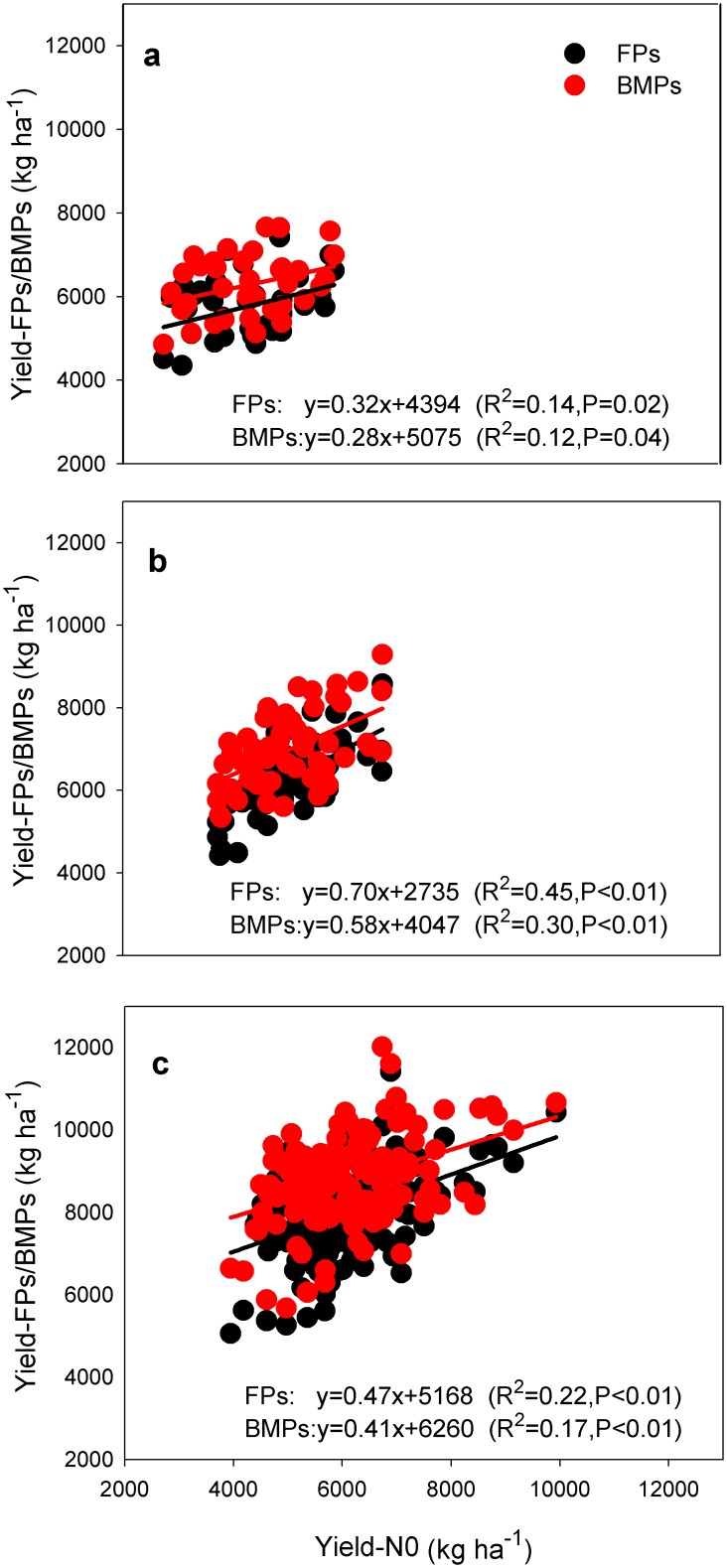
Relationships among management practices, soil inherent productivity and yield. Note: soil inherent productivity was estimated as yields in zero-N plots (Yield-N0). Black points represent farming practice (FPs); red points represent best management practice (BMPs). a, early rice (n = 98); b, late rice (n = 148); c, single rice (n = 157).

The rice yields in 403 on-farm trials conducted on soils with different inherent productivities across the major rice farming systems were on average 5998 kg ha^-1^ for early rice, 6370 kg ha^-1^ for late rice, and 8305 kg ha^-1^ for single rice across all sites and management practices (FPs and BMPs). Rice yields under both FPs and BMPs showed significant positive relationships with yield in zero-N plots (Fig 1). This suggested that inherently more fertile soils produced higher crop yields than the poorer soils irrespective of the management practices employed.

**Fig 2:**
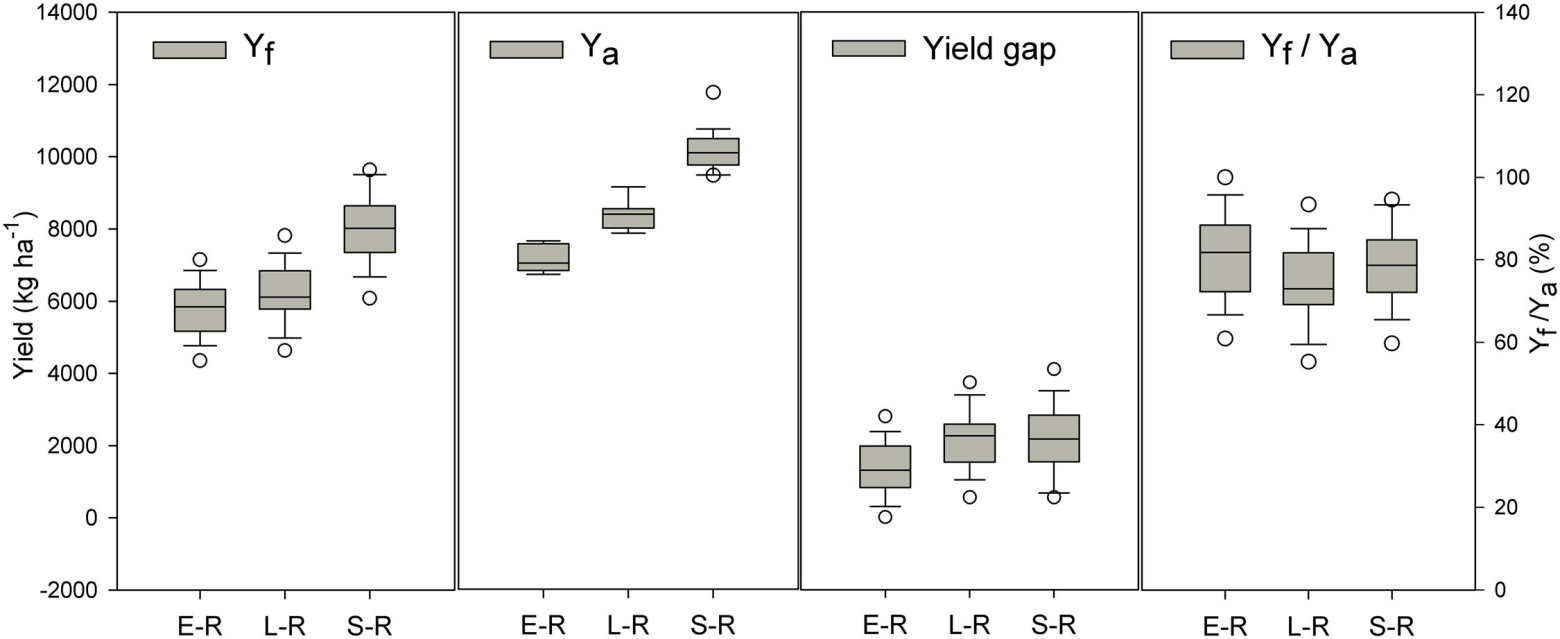
Yield of farming practices (Y_f_), attainable yield (Y_a_), yield gap and the percentage of Y_f_ as Ya (Y_f_ /Y_a_) for three rice faming systems. Note: yield gaps were estimated as differences between yields in farming practice on soils with various productivity levels and ‘attainable yields’, determined as mean yields of the 20% highest-yielding locations under BMPs. E-R, early rice, L-R, late rice, S-R, single rice. Solid and dashed lines indicate median and mean yields, respectively. The box boundaries indicate upper and lower quartiles, the whisker caps indicate 90th and 10th percentiles, and the circles indicate the 95th and 5th percentiles.

**Fig 3:**
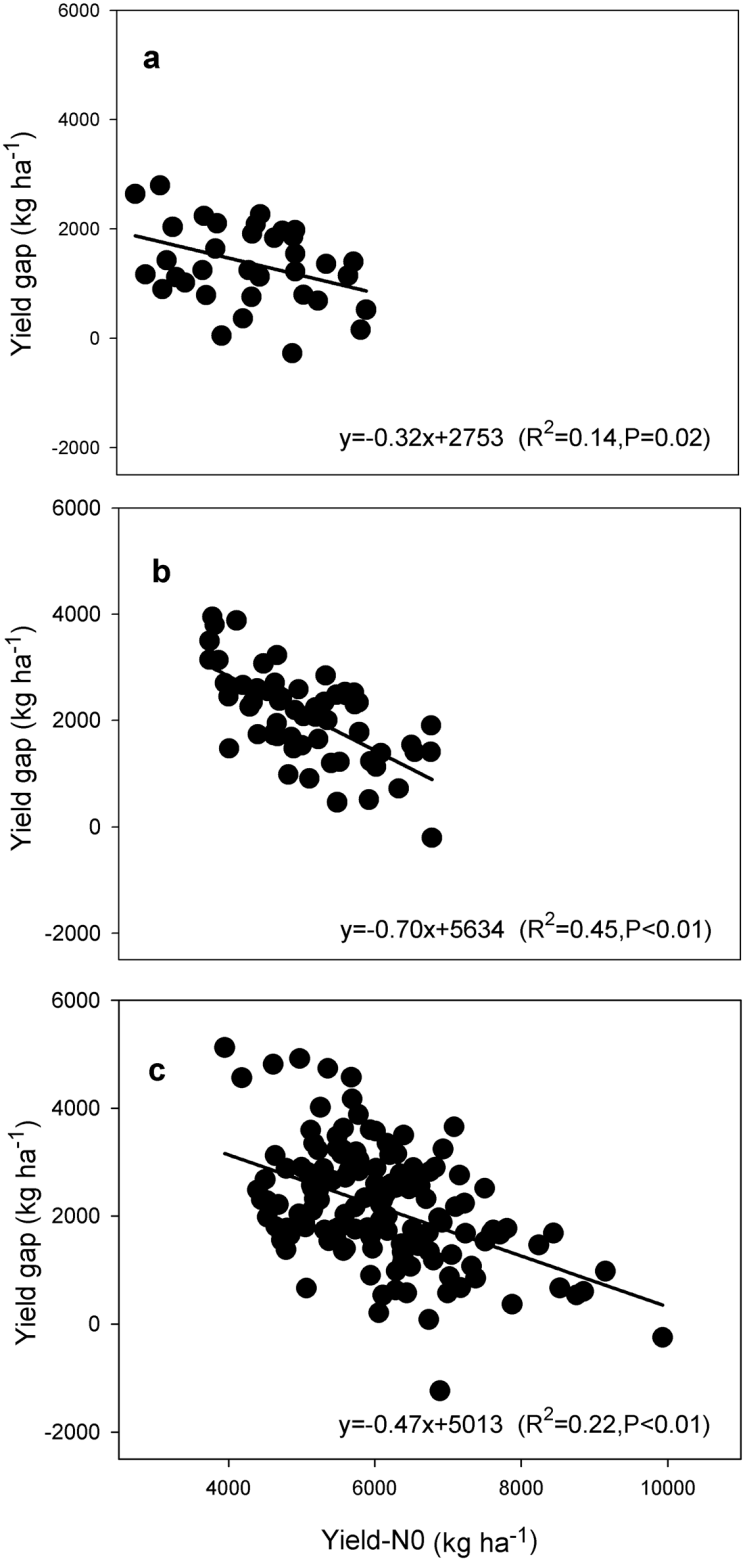
Relationships among management practices, soil inherent productivity and yield gap. Note: soil inherent productivity was estimated as yield in zero-N plots (Yield-N0). Yield gaps were estimated as differences between yields in farming practice on soils with various productivity levels and ‘attainable yields’, determined as mean yields of the 20% highest-yielding locations under BMPs. (a), early rice (n = 98); (b), late rice (n = 148); (c), single rice (n = 157).

The average yield of FPs was 81% of the average attainable yield for early rice, 75% for late rice, and 79% for single rice (Fig 2). The average yield gaps were 1401, 2375 and 2282 kg ha^-1^ with ranges from 0 to 2798 kg ha^-1^ for early rice, 0 to 3949 kg ha^-1^ for late rice, and 0 to 5121 kg ha^-1^ for single rice production systems across all locations (Fig 2). In contrast to crop yield, the yield gap was negatively correlated with the yield from zero-N plots for all three rice types (Fig 3a-3c).

### Interactive effects of soil inherent productivity, rice farming type and management practices on N_2_O and CH_4_ emissions, global warming potential and greenhouse gas intensity

**Fig 4:**
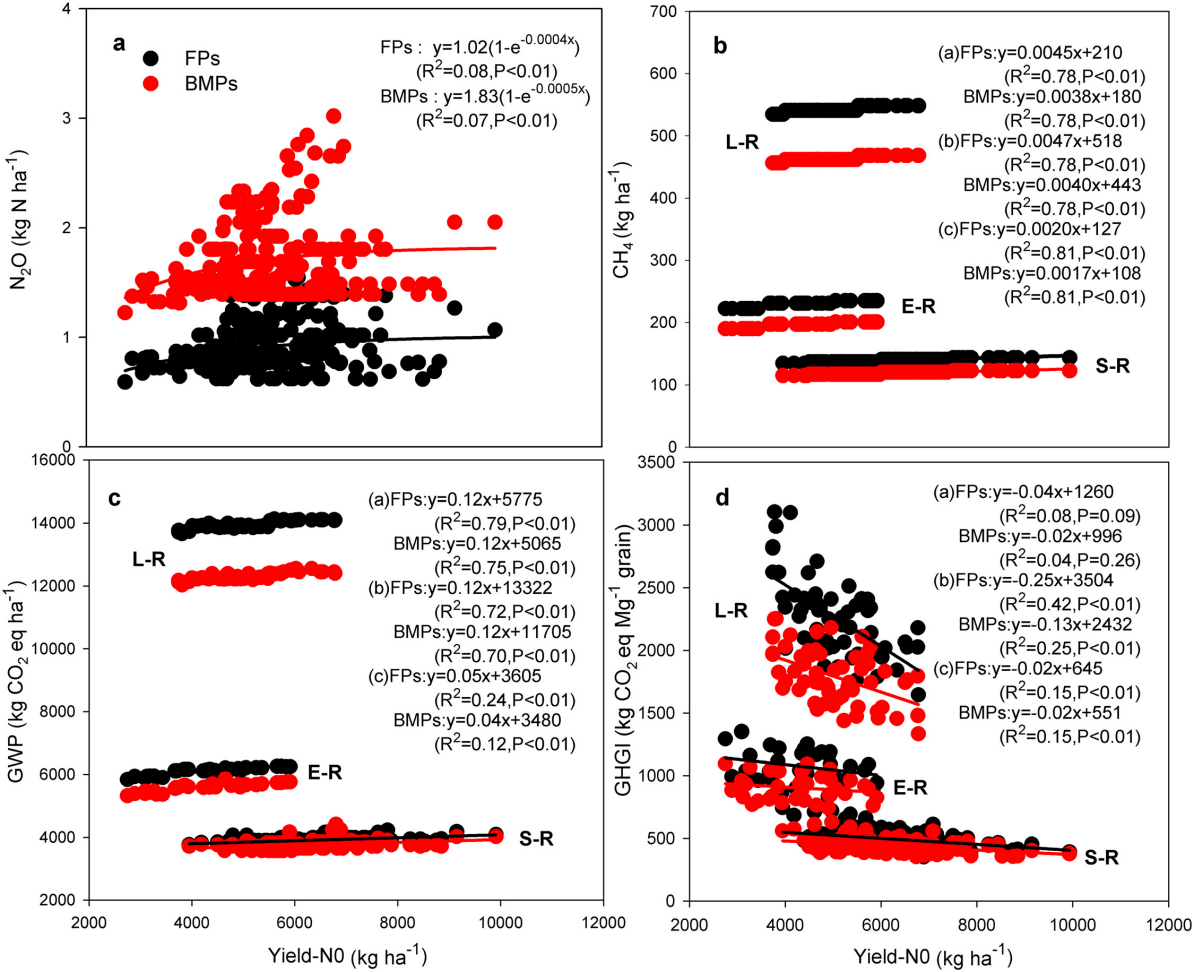
Relationships among management practices, soil inherent productivity and N_2_O emissions (a), CH_4_ emissions (b), global warming potential (GWP, c), and greenhouse gas emission intensity (GHGI, d). Note: soil inherent productivity was estimated as yield in zero-N plots (Yield-N0). The GWP and GHGI is the sum of emissions of CO_2_-eq of N_2_O and CH_4_ at area and yield scale during the rice growing season, respectively. Black points represent farming practice (FPs); red points represent best management practice (BMPs). For b, c and d, L-R, E-R and S-R represent late rice (n = 148), early rice (n = 98) and single rice (n = 157).

For both FPs and BMPs, N_2_O emissions estimated as a function of N rate (S2 Fig F-D-F for FPs and F-D-F-M for BMPs) increased exponentially with increasing inherent soil productivity (Fig 4a). Emissions of CH_4_ differed significantly among rice production systems in the sequence late > early > single rice (Fig 4b). Positive relationships were observed between CH_4_ emissions and inherent soil productivity for both FPs and BMPs (Fig 4b). GWP also slightly increased with increasing inherent soil productivity (Fig 4c). However, GHGI decreased with increasing soil productivity and was significant for late and single rice systems (Fig 4d). Therefore, cultivating soils of high inherent productivity increased CO_2_-eq emissions per unit area but decreased CO_2_-eq emissions of N_2_O and CH_4_ per unit of yield. Similar to emissions of CH_4_, GWP and GHGI also followed the order late > early > single rice systems (Fig 4c and 4d).

The average N_2_O emissions were consistently higher in BMPs than those in FPs irrespective of soil inherent productivity (Fig 4a). In contrast, adoption of BMPs reduced CH_4_ emissions, GWP and GHGI especially in the double rice production systems (Fig 4b-4d).

### Yield, N rate and greenhouse gas emission responses under best management practices

Adoption of BMPs led to higher yield responses in late and single rice systems than those in early rice. Compared to FPs, average yield increases from BMPs in 403 on-farm trials were 512, 757 and 733 kg ha^-1^ for early, late and single rice, respectively. As soil productivity increased the BMP responses in yield showed decreasing trends (Table 1). A negative response in N rate shows that BMPs consistently led to lower fertilizer N application than FPs. Over all soil inherent productivity levels and rice farming systems, N fertilizer rate upon adoption of BMPs could be reduced by 35 kg N ha^-1^ compared with FPs, with the greatest reduction in absolute terms for single rice and as a percentage for early rice systems (Table 1).

**Table 1:** Responses in rice yield, N rate, N_2_O, CH_4_, global warming potential (GWP) and greenhouse gas intensity (GHGI) of the best management practices (BMPs) adopted on soils of different inherent productivity levels for early and late rice in south China and single rice in the Yangtze Delta.

Inherent soil productivity^†^	Response of BMPs^‡^																	
	ΔYield (kg ha^-1^)			ΔN rate (kg N ha^-1^)			ΔN_2_O (kg N ha^-1^)			ΔCH_4_ (kg ha^-1^)			ΔGWP^§^ (kg CO_2_-eq ha^-1^)			ΔGHGI^§^ (kg CO_2_-eq Mg^-1^ grain)		
	Early	Late	Single	Early	Late	Single	Early	Late	Single	Early	Late	Single	Early	Late	Single	Early	Late	Single
d_1_	557	892	853	-35	-28	0	0.72	0.74	0.77	-32.9	-78.1	-19.6	-505	-1631	-136	-189	-655	-76
d_2_	506	739	767	-31	-38	-48	0.78	0.79	0.80	-33.8	-79.0	-20.1	-502	-1636	-140	-165	-486	-62
d_3_	474	640	689	-33	-31	-59	0.80	0.81	0.82	-34.3	-79.6	-20.4	-501	-1640	-143	-151	-407	-52
d_4_	——	——	623	——	——	-54	——	——	0.82	——	——	-20.8	——	——	-146	——	——	-45
Mean	512*	757*	733*	-33*	-32*	-40*	0.77*	0.78*	0.80*	-33.7*	-78.9*	-20.2*	-503*	-1636*	-141*	-168*	-516*	-59*

*Note*:

^†^Inherent soil productivity estimated as yields in zero-N plots; d_1_, d_2_, d_3_ and d_4_ refer to inherent soil productivity grades (see SI materials and methods on classification);

^‡^ Response of BMPs defined as difference in yield, N rate and greenhouse gas emissions between BMPs and current farming practice, the on-farm trails involved in analysis were 98 for early, 148 for late and 157 for single rice systems, respectively.

^§^ GWP and GHGI is the sum of emission of CO_2_-eq of N_2_O and CH_4_ at area and yield scale during rice growing season, respectively.

* Present significant differences between BMPs and current farming practices over all locations at P = 0.05 level.

In contrast to N_2_O response pattern, the inherently better soils seem to lead to higher reduction in CH_4_ emissions and GWP. Average BMP responses in terms of CH_4_ emissions and GWP followed the order late > early > single rice systems, showed varying potential in mitigating climate changes upon adopting BMPs (Table 1).

The mean GHGI changes showed patterns similar to those of GWP and were -516 kg CO_2_-eq Mg^-1^ grain for late rice, -168 kg CO_2_-eq Mg^-1^ grain for early rice, and -59 kg CO_2_-eq Mg^-1^ grain for single rice. However, within each cropping system the change in GHGI decreased with an increase in inherent soil productivity (Table 1).

### Total rice production, fertilizer N consumption and N_2_O, CH_4_ and global warming potential under alternative strategies

The total rice production, fertilizer N consumption, and GHG emissions under alternative strategies were evaluated (Table 2). Compared to continuing the FP scenario, adoption of BMPs across the major single and double cropping systems would increase rice production in China by 16.9 × 10^6^ Mg (10%). Despite a decrease in total fertilizer N consumption by 1.0 × 10^6^ Mg (20%), adopting BMPs also nearly doubled the increase in N_2_O emissions compared with FPs. However, the increase in N_2_O was offset by a 15% decrease in CH_4_ emissions and consequently led to a reduction of CO_2_-eq emissions by 14.9 × 10^6^ Mg (9%).

**Table 2:** Total rice production, N fertilizer consumption and greenhouse gas emissions (N_2_O, CH_4_ and GWP) across Chinese major rice farming systems following different strategies.

Scenario*	Total rice production		Total fertilizer N consumption		Total N_2_O emissions		Total CH_4_ emissions		GWP of N_2_O and CH_4_	
	Production (10^6^ Mg)^†^	Increase^‡^ (%)	Fertilizer N consumption (10^6^ Mg)^†^	Increase (%)	N_2_O (10^3^ Mg)^†^	Increase (%)	CH_4_ (10^4^ Mg)^†^	Increase (%)	GWP^§^ (10^6^ Mg CO_2_-eq)^†^	Increase (%)
FPs	169.3	——	4.82	——	21.4	——	642.1	——	170.3	——
Adopting BMPs	186.3	10.0	3.86	-20.0	40.8	90.6	548.2	-14.6	155.4	-8.7
Increasing ISP and FPs	185.4	9.5	4.83	0.1	22.8	6.2	652.1	1.5	172.8	1.5
Adopting BMPs and Increasing ISP	200.0	18.1	3.75	-22.1	42.6	98.8	556.7	-13.3	157.9	-7.3

*Note*:

*FPs, current farming practice continues; Adopting BMPs, adopting best management practices such as improved N and water management, cultivating healthy seedlings and increasing rice transplanting density; Increase ISP and FPs, increasing inherent soil productivity by 1500 kg ha^-1^ for soils of low and moderate level but with adoption of current farming practice; and Adopting BMPs and Increasing ISP, a combination of adopting best management practices and increasing inherent soil productivity.

^†^Total rice production, fertilizer N consumption and greenhouse gas emissions is the sum of those in early and late rice in south China and single rice in the Yangtze Delta (see SI materials and methods on calculation).

^‡^ Refer to increase in relative percentages with adopting BMPs, increase SIP and FPs or adopting BMPs and Increasing ISP, compared with FPs.

^§^GWP is the sum of emission of CO_2_-eq of N_2_O and CH_4_ during rice growing season.

A strategy of increasing inherent soil productivity by 1500 kg ha^-1^ for soils of low and moderate inherent productivity but with continued use of FPs could increase rice production by 16.1 × 10^6^ Mg (10%) compared with continuing current management practices. However, this would lead to a small increase in fertilizer N consumption of 0.01 × 10^6^ Mg (0.1%) and GHG emissions (6.2% for N_2_O, 1.5% for CH_4_ and 2% for GWP).

The greatest benefit will be obtained by the combined adoption of BMPs and increased inherent soil productivity, leading to increases in total rice production (compared with continuing FPs) by 18%, with reductions in fertilizer N consumption and emissions of CO_2_-eq of 22 and 7% (Table 2).

Compared to the scenario of continuing FPs, single rice would account for on average 53% of the total increases in rice production upon following new alternative strategies such as adoption of BMPs, increasing soil inherent productivity or a combination of both; the early and late rice together account for 47% of total rice production (S1 Table). Single rice could account for about a 58–65% reduction in fertilizer N consumption with adoption of the BMPs or a combination of adopting BMPs and increasing soil inherent productivity and late and early rice together could account for about 35–42% reduction in fertilizer N consumption, respectively. The contribution of late and early rice to reduction in CO_2_-eq emissions accounts for about 69–75% and 17–20% with adoption of the BMPs or a combination of the BMPs and increasing soil inherent productivity. However, the contribution of single rice to reduced CO_2_-eq emissions during the rice growth period was small to negligible (S1 Table).

## Discussion

### Exploiting co-benefits of increased rice productivity and production with reduced greenhouse gas emission through optimized crop and soil management

Closing yield gaps through improved management has been suggested as key strategy for increasing crop production on limited areas of arable land [34–36]. Based on 403 on-farm trials, we show that compared to FPs, a few carefully chosen BMPs could increase rice yields by 474–892 kg ha^-1^ across all locations and rice farming systems (Table 1). This confirms and greatly expands on the results from more technology-specific studies conducted in recent years in China and other countries [21,23,29,37]. Genetic improvements are another key measure to take in conjunction with that because new varieties need to be well adapted to changing environments and market requirements. The yield increase with adoption of BMPs may be through (1) increased transplanting density and the attendant high-yielding rice population [38], (2) N application in accordance with the physiological N requirements of the crop [22,39,40], (3) balanced application of P and K [29,41], and (4) alternate dry and wet irrigation after midseason drainage with the attendant enhancement of root systems and grain filling [42,43].

Despite a reduction in N fertilizer application, BMPs led to increased N_2_O emissions. In contrast to N_2_O emissions, adoption of BMPs reduced CH_4_ emissions (Table 1; Fig 4a and 4b). Clearly, water management was a major factor in the contrasting effects on CH_4_ and N_2_O emissions between FPs and BMPs [15,16]. The higher GWP and GHGI under FPs than BMPs, especially for late and early rice in south China (Fig 4c and 4d; Table 1), implies that reducing CH_4_ emissions can offset the increased N_2_O emissions for changing from FPs to BMPs.

Adoption of BMPs across the major rice cropping systems in south China and the Yangtze Delta might increase total rice production by 10%, reduce fertilizer N use by 20% and reduce CO_2_-eq emissions by 9% compared with strategies that continue past trends (Table 2). It is estimated that in order to double crop production worldwide global fertilizer N use in 2050 would be approximately 250 × 10^6^ Mg yr^-1^ [2], an increase of 140% compared to the 104 × 10^6^ Mg applied in 2010 [44]. The data from the current study show that increasing Chinese rice production is possible despite the reduction in fertilizer N use by 20%. Similar conclusions were reached in global analysis [45]–provided that a decline in cereal harvested can be halted.

However, with adoption of BMPs alone increases in total rice production and average yield per unit area across the rice farming systems in the current study would probably not meet the projected demand of a 20% increase in total production [46] or 20–23% increase in yield per unit area by 2030 [1]. This signifies the difficulty in closing yield gaps by adoption of better crop, nutrient and water management in the major Chinese rice production systems where average yields in farmers’ fields already approach 70% or more of the attainable yields and the marginal return from improving management practices becomes progressively smaller [34,47].

An important finding in our study—based on farm-level data—was that yields under BMPs were positively and yield gaps negatively correlated with the inherent soil productivity (Figs 1 and 3). In view of the small yield responses to BMPs (474–892 kg ha^-1^), the wide range in yield gaps (0 to 5121 kg ha^-1^) suggests that in addition to management practices, inherent soil productivity may be a principal determinant of the yield gap, especially on soils with relatively low productivity (Fig 3). Furthermore, due to the fact that most of the paddy soils in south China and the Yangtze Delta belong to the low and intermediate productivity categories (Table D in S1 Text), it is difficult for a large number of rice farmers to close the yield gap without an increase in inherent soil productivity. A strategy of combining improved low- and moderate-yielding farmland and adoption of BMPs might narrow yield gap and lead to the majority of farmers achieve yields of > 85% of the attainable yield and with efficient use of resources. Such a strategy, if adopted across all of the major cropping systems, would increase rice production by 18% with reductions in fertilizer N consumption and emissions of CO_2_-eq of 22 and 7% (Table 2). The 18% increase in total production for single rice in the Yangtze Delta and double rice in the south would be more than adequate to meet the demand for rice for direct human consumption in China by 2030. It should be noted that rice farming systems in other regions of the country have not been involved in the current study and may also contribute about 20% to the total rice production nationally [28].

BMPs, such as increasing transplanting density, reducing the N application rate and applying a larger proportion of the N at intermediate growth stages, balancing application rates of P and K, and shifting water management from F-D-F to F-D-F-M represent practices that—with sufficient knowledge dissemination, training and other investments—can be applied widely in rice cropping systems. For example, the use of low-cost transplanting machines could alleviate the problem of the low transplanting densities of current farming practices [48]. Adoption of F-D-F-M irrigation has been promoted in Chinese rice production because of water shortages and the development of cultivation techniques. The use of the F-D-F-M irrigation pattern increased from 7% of paddy soils in the 1980s to 12% in the 1990s [49]. National and international collaboration has already led to the development and widespread on-farm validation of easily applied integrated and site-specific nutrient management approaches [23,30].

Despite the great variation in inherent soil productivity within each China’s rice domains, inherent better soil productivity are linked to relative higher average SOC and total nitrogen concentration (Table D in S1 Text). This may be a result of differences in management practices and yields during the past few decades [33,50]. Further, it has also been found that various forms of soil degradation such as thin and compacted topsoil and soil acidification remain widespread across Chinese paddy soils [51–54]. Thus, there remains an opportunity to increase inherent productivity further, especially of soils with low or moderate productivity. Other interventions include consolidating small land holdings, leveling soil, improving irrigation conservation facilities.

Thus, though uncertainty persists (S2 Text as Supplementary discussions), the current study provide on-farm evidences toward increase rice productivity and production with less environmental impacts. The future priority lies in national coordination and a multidisciplinary approach to promote adoption of BMPs and increasing soil productivity simultaneously. Both the public and private sector will need to find new ways for effectively working together on that in order to bring real progress to millions of rice farmers in China.

It should also be noted that cultivating soils of higher productivity may imply much higher N_2_O and CH_4_ emissions and consequently higher GWP than low productivity soils, even under BMPs (Fig 4a-4c). The higher N_2_O emissions from soils with higher productivity may be due to higher rates of N fertilizer application and the higher CH_4_ emissions may be attributed to the higher SOC concentrations in soils of high inherent productivity (Table D in S1 Text) providing methanogenic substrates [55] and contributing to lower soil Eh conditions [56]. However, the advantages of lower inputs and smaller environmental footprints of low productivity soils disappear when the metrics are scaled by grain yield (Fig 4d). Further, the extra benefit is that increasing soil productivity on land already under agriculture constitutes a clear opportunity to increase yields per unit area and avoid further land clearance, and thus reduces GHG emissions and species extinctions that would otherwise have resulted from land clearance [57–60].

### The roles of rice farming types in further increasing rice productivity and production with lower greehouse gas emission

The specific types of rice farming systems which are practiced over a wide range of climate zones may primarily reflect the effects of climatic conditions (e.g. growth period and solar radiation intensity) on rice production [27,61] and indicate varying potential to enhance agronomic yields and reduce the environmental footprint.

Single rice in the Yangtze Delta produced higher yields due to its longer growth periods than either early or late rice only in south of China (Fig 1). However, double rice system led to higher total yield and yield response increase upon adopting BMPs than single rice systems. As a result of the wider yield gap and total cultivated area of single rice Yangtze Delta than double rice in south of China (Fig 2 and Table D in S1 Text), the former has more potential in total rice production by following the new alternative strategies such as adoption of BMPs, increasing inherent soil productivity or some combination of both (S1 Table). However, single rice systems showed negligible reduction in GWP upon adoption of new alternative strategies (S1 Table). Because of its higher CH_4_ emissions, double rice (and especially late rice) has greater GWP than single rice systems irrespective of management practices (Fig 4b and 4c). Due to the higher response to BMPs in reducing the GWP, late rice showed a higher reduction in total emissions followed by early rice upon adoption of BMPs or a combination of BMPs and increasing soil productivity (S1 Table). These findings leave land managers with important choices in terms of which cropping type offers the best hope of meeting projected rice production demands and where the best locations for reducing the environmental impact of rice production are.

## Conclusion

In summary, the study indicates confounded and interactive effects of inherent soil productivity, management practices and rice farming types on agronomic productivity and major GHG emissions of rice farming systems. There is considerable potential for closing the yield gap with less N and water use and reducing the environmental footprint. However, this requires improvements in both management practices and inherent soil productivity across the major rice farming systems to harness the combined benefits of production enhancement and mitigating climate change.

In a broad context, the current study is a bench mark for integration of agronomic and environmental analysis of practical management options and soil productivity at the sub-cropping systems scale. The study provides the basis for re-orientation and identification of feasible and practical approaches and management practices in meeting concerns over food security and environmental quality worldwide.

## Supporting Information

S1 Fig Geographical distribution of the dataset. a, 403 on-farm trials conducted on soils with various inherent productivities in the major Chinese rice cropping systems from 2008–2011; b, Yield data in zero-N conditions derived from 5351 locations for assessment of inherent soil productivity of major rice farming systems.
(DOC)

S2 Fig Relationships between nitrogen fertilizer rate and N_2_O emissions in paddy for traditional continuous flooding (F-D-F) and intermittent irrigation after midseason drainage (F-D-F-M) in south China and the Yangtze Delta. Black points represent F-D-F (n = 51) and white points represent F-D-F-M (n = 44).
(DOC)

S3 Fig Relationships in rice yield between zero-N (Yield-N0) and fertilizer-omission plots (Yield-CK). Dataset derived from 5351 locations. Early rice (n = 861), late rice (n = 1055), single rice (n = 3435) and rice (n = 5351).
(DOC)

S1 Table Total rice production, fertilizer N consumption and global warming potential (GWP) of N_2_O and CH_4_ following different strategies for early and late rice in south China and single rice in the Yangtze Delta. (DOC)

S1 Text Supplementary materials and methods. (DOC)

S2 Text Supplementary discussions. (DOC)

## References

[1] Dobermann A, Nelson R, Beever D, Bergvinson D, Crowley E, Denning G, et al Solutions for sustainable agriculture and food systems Technical report for the post-2015 development agenda. New York: Sustainable Development Solutions Network; 2013.

[2] Tilman D, Balzer C, Jason H, Befort BF. Global food demand and the sustainable intensification of agriculture. Proc Natl Acad Sci USA. 2011; 108(50): 20260–20264. 10.1073/pnas.1116437108 22106295 PMC3250154

[3] Pandey S, Byerlee D, Dawe D, Dobermann A, Mohanty S, Rozelle S, et al Rice in the global economy: strategic research and policy issues for food security. Los Banos: International Rice Research Institute; 2010.

[4] Chen HZ, Zhu DF, Yang SH, Zhang YP, Lin XQ. The difference and potential of rice yield in southern China. China Rice. 2004; 4: 9–10 (in Chinese with English abstract).

[5] Fan MS, Shen JB, Yuan LX, Jiang RF, Chen XP, Davies WJ, et al Improving crop productivity and resource use efficiency to ensure food security and environmental quality in China. J Exp Bot. 2012; 63(1): 13–24. 10.1093/jxb/err248 21963614

[6] Laborte AG, De Bie K, Smaling EMA, Moya PF, Boling AA, Van Ittersum MK. Rice yields and yield gaps in Southeast Asia: past trends and future outlook. Eur J Agron. 2012; 36(1): 9–20.

[7] Mueller ND, Gerber JS, Johnston M, Ray DK, Ramankutty N, Foley JA. Closing yield gaps through nutrient and water management. Nature. 2012; 490: 254–257. 10.1038/nature11420 22932270

[8] Ray DK, Ramankutty N, Mueller ND, West PC, Foley JA. Recent patterns of crop yield growth and stagnation. Nat Commun. 2012; 3: 1293 10.1038/ncomms2296 23250423

[9] Grassini P, Eskridge KM, Cassman KG. Distinguishin between yield advances and yield plateaus in historical crop production trends. Nat Commun. 2013; 4: 2918 10.1038/ncomms3918 24346131 PMC3905725

[10] Chen XP, Cui ZL, Fan MS, Vitousek P, Zhao M, Ma WQ, et al Producing more grain with lower environmental costs. Nature. 2014; 514: 486–489. 10.1038/nature13609 25186728

[11] Heffer P. Assessment of fertilizer use by crop at the global level: 2006/07–2007/08. Paris: International Fertilizer Industry Association; 2009.

[12] FAO Statistical Yearbook. Rome: Food and Agriculture Organization of the United Nations; 2013 pp. 160.

[13] Bouman BAM, Humphreys E, Tuong TP, Barker R. Rice and water. Adv Agron. 2007; 92: 187–237.

[14] Tian J, Fan MS, Lv SH, Li XL, Kuzyakov Y. Integrated management systems and N fertilization: effect on soil organic matter in rice-rapeseed rotation. Plant Soil. 2013; 372(1–2): 53–63.

[15] Cai ZC, Xing GX, Yan XY, Xu H, Tsuruta H, Yagi K, et al Methane and nitrous oxide emissions from rice paddy fields as affected by nitrogen fertilisers and water management. Plant Soil. 1997; 196(1): 7–14.

[16] Li CS, Frolking S, Xiao XM, Moore B III, Boles S, Qiu JJ, et al Modeling impacts of farming management alternatives on CO_2_, CH_4_, and N_2_O emissions: a case study for water management of rice agriculture of China. Global Biogeochem Cycles. 2005; 9(3): 1–10.

[17] Zou JW, Huang Y, Jiang JY, Zheng XH, Sass RL. A 3-year field measurement of methane and nitrous oxide emissions from rice paddies in China: effects of water regime, crop residue, and fertilizer application. Global Biogeochem Cycles. 2005; 19(2): GB2021, 10.1029/2004GB002401

[18] Pittelkow CM, Adviento-Borbe MA, Hill JE, Six J, Kessel CV, Linquist BA. Yield-scale global warming potential of annual nitrous oxide and methane emissions from continuously flooded rice in response to nitrogen input. Agric Ecosyst Environ. 2013; 177: 10–20.

[19] Yan XY, Yagi K, Akiyama H, Akimoto H. Statistical analysis of the major variables controlling methane emission from rice fields. Global Change Biol. 2005; 11(7): 1131–1141.

[20] Pittelkow CM, Adviento-Borbe MA, Kessel CV, Hill JE, Linquist BA. Optimizing rice yields while minimizing yield-scaled global warming potential. Global Change Biol. 2014; 20(5): 1382–1393. 10.1111/gcb.12413 24115565

[21] Wang GH, Dobermann A, Witt C, Sun QZ, Fu RX. Performance of site-specific nutrient management for irrigated rice in southeast China. Agron J. 2001; 93(4): 869–878.

[22] Fan MS, Lv SH, Jiang RF, Liu XJ, Zhang FS. Triangular transplanting pattern and split nitrogen fertilizer application increase rice yield and nitrogen fertilizer recovery. Agron J. 2009; 101(6): 1421–1425.

[23] Peng SB, Buresh RJ, Huang JL, Zhong XH, Zou YB, Yang JC, et al Improving nitrogen fertilization in rice by site-specific N management. A review. Agron Sustain Dev. 2010; 30(3): 649–656.

[24] Ma YC, Kong XW, Yang B, Zhang XL, Yan XY, Yang JC, et al Net global warming potential and greenhouse gas intensity of annual rice-wheat rotations with integrated soil-crop system management. Agric Ecosyst Environ. 2013; 164: 209–219.

[25] Patzel N, Sticher H, Karlen DL. Soil fertility-phenomenon and concept. J Plant Nutr Soil Sc. 2000; 163(2): 129–142.

[26] Bai ZG, Dent DL, Olsson L, Schaepman ME. Global assessment of land degradation and improvement 1. Identification by remote sensing. Wageningen: ISRIC- World Soil Information; 2008.

[27] Maclean JL, Daw DC, Hardy B, Hettel GP. Rice almanac. Wallingford: CABI Publishing; 2002.

[28] China Statistical Yearbook. Beijing: National Bureau of Statistics of China. China Statistics Press 2011; Available: http://data.stats.gov.cn/publish.

[29] Dobermann A, Witt C, Dawe D, Abdulrachman S, Gines GC, Nagarajan R, et al Site-specific nutrient management for intensive rice cropping systems in Asia. Field Crop Res. 2002; 74(1): 37–66.

[30] Zhang FS, Cui ZL, Chen XP, Ju XT, Shen JB, Chen Q, et al Integrated nutrient management for food security and environmental quality in China. Advan Agron. 2012; 116: 1–32.

[31] Zhang H, Zhang SF, Yang JC, Zhang JH, Wang ZQ. Postanthesis moderate wetting drying improves both quality and quantity of rice yield. Agron J. 2008; 100(3): 726–734.

[32] Dobermann A, Fairhurst TH. Rice: nutrient disorders and nutrient management Singapore: Potash and Phosphate Institute. Manila: IRRI; 2000.

[33] Fan MS, Lal R, Cao J, Qiao L, Jiang RF, Zhang FS. Plant-based assessment of inherent soil productivity and contributions to China’s cereal crop yield increase since 1980. PLoS ONE. 2013; 8(9): e74617 10.1371/journal.pone.0074617 24058605 PMC3776784

[34] Cassman KG. Ecological intensification of cereal production systems: yield potential, soil quality, and precision agriculture. Proc Natl Acad Sci USA. 1999; 96(11): 5952–5959. 10339523 10.1073/pnas.96.11.5952 PMC34211

[35] Foley JA, Ramankutty N, Brauman KA, Cassidy ES, Gerber JS, Johnston M, et al Solutions for a cultivated planet. Nature. 2011; 478(7369): 337–342. 10.1038/nature10452 21993620

[36] George T. Why crop yields in developing countries have not kept pace with advances in agronomy. Global Food Security. 2014; 3(1): 49–58.

[37] Pampolino MF, Manguiat IJ, Ramanathan S, Gines HC, Tan PS, Chi TTN, et al Environmental impact and economic benefits of site-specific nutrient management (SSNM) in irrigated rice systems. Agric Syst. 2007; 93(1–3): 1–24.

[38] An N, Fan MS, Zhang FS. Increase both rice yield and nitrogen use efficiency by the best crop management practices. Plant Nutr Fert Sci. 2015; 21(4): 846–852 (in Chinese with English abstract).

[39] Perez CM, Juliano BO, Liboon SP, Alcantara JM, Cassman KG. Effects of late nitrogen fertilizer application on head rice yield, protein content, and grain quality of rice. Cereal Chem. 1996; 73(5): 556–560.

[40] Cassman KG, Peng SB, Olk DC, Ladha JK, Reichardt W, Dobermann A, et al Opportunities for increased nitrogen use efficiency from improved resource management in irrigated rice systems. Field Crop Res. 1998; 56(1–2): 7–39.

[41] Buresh RJ, Pampolino MF, Witt C. Field-specific potassium and phosphorus balances and fertilizer requirements for irrigated rice-based cropping systems. Plant Soil. 2010; 335(1–2): 35–64.

[42] Bouman BAM. A conceptual framework for the improvement of crop water productivity at different spatial scales. Agric Syst. 2007; 93(1–3): 43–60.

[43] Zhang H, Chen TT, Wang ZQ, Yang JC, Zhang JH. Involvement of cytokinins in the grain filling of rice under alternate wetting and drying irrigation. J Exp Bot. 2010; 61(13): 3719–3733. 10.1093/jxb/erq198 20584789

[44] IFA Database. Paris: International Fertilizer Industry Association. 2012; Available: http://www.fertilizer.org.

[45] Dobermann A, Cassman KG. Cereal area and nitrogen use efficiency are drivers of future nitrogen fertilizer consumption. Sci China Ser C. 2005; 48: 745–758. 10.1007/BF03187115 20549431

[46] Peng SB, Tang QY, Zou YB. Current status and challenges of rice production in China. Plant Prod Sci. 2009; 12(1): 3–8.

[47] Wart JV, Kersebaum KC, Peng SB, Milner M, Cassman KG. Estimating crop yield potential at regional to national scales. Field Crop Res. 2013; 143: 34–43.

[48] Wu LQ, Cai GX, Shi XJ, Chen XP. The agronomical effects of formulated fertilizer and mechanical transplanting of rice. China Agric Tech Exten. 2013; 29(1): 35–36 (in Chinese).

[49] Zou JW, Huang Y, Qin YM, Liu SW, Shen QR, Pan GX, et al Changes in fertilizer-induced direct N2O emissions from paddy fields during rice-growing season in China between 1950s and 1990s. Global Change Biol. 2009(1); 15: 229–242.

[50] Dobermann A, Witt C, Abdulrachman S, Gines HC, Nagarajan R, Son TT, et al Soil fertility and indigenous nutrient supply in irrigated rice domains of Asia. Agron J. 2003; 95(4): 913–923.

[51] Lindert PH. Shifting ground: the changing agricultural soils of China and Indonesia. Cambridge: MIT Press; 2000.

[52] Chen J, Chen JZ, Tan MZ, Gong ZT. Soil degradation: a global problem of endangering sustainable development. J Geogr Sci. 2002; 12(2): 243–252.

[53] Peng SQ, Tian YG, Zhong YH, Ma CB, Sun Z, Ren Y. Research report on soil degradation and governance in major grain producing provinces of China. China Agric Tech Exten. 2006; 22: 280–291 (in Chinese).

[54] Guo JH, Liu XJ, Zhang Y, Shen JL, Han WX, Zhang WF, et al Significant acidification in major Chinese croplands. Science. 2010; 327(5968): 1008–1010. 10.1126/science.1182570 20150447

[55] Holzapfel-Pschorn A, Seiler W. Methane emission during a cultivation period from an Italian rice paddy. J Geophys Res. 1986; 91(D11): 11803–11814.

[56] Neue HU, Roger PA. Potential of methane emission in major rice ecologies In: Zepp RG, editor. Climate biosphere interaction: biogenic emissions and environmental effects of climate change. New York: John Wiley and Sons; 1994 pp. 65–93.

[57] Green RE, Cornell SJ, Scharlemann JPW, Balmford A. Farming and the fate of wild nature. Science. 2005; 307(5709): 550–555. 15618485 10.1126/science.1106049

[58] Burney JA, Davis SJ, Lobell DB. Greenhouse gas mitigation by agricultural intensification. Proc Natl Acad Sci USA. 2010; 107(26): 12052–12057. 10.1073/pnas.0914216107 20551223 PMC2900707

[59] West PC, Gibbs HK, Monfreda C, Wagner J, Barford CC, Carpenter SR, et al Trading carbon for food: global comparison of carbon stocks vs. crop yields on agricultural land. Proc Natl Acad Sci USA. 2010; 107(44): 19645–19648. 21041633 10.1073/pnas.1011078107 PMC2993385

[60] Phalan B, Onial M, Balmford A, Green RE. Reconciling food production and biodiversity conservation: land sharing and land sparing compared. Science. 2011; 333(6047): 1289–1291. 10.1126/science.1208742 21885781

[61] Gao LZ, Guo P, Zhang LZ, Lin W. Light and heat resources and potential productivity of rice in China. Sci Agric Sin. 1984; 1: 17–23 (in Chinese with English abstract).

