# Supplementary material for: Exploiting Co-Benefits of Increased Rice Production and Reduced Greenhouse Gas Emission through Optimized Crop and Soil Management
Source: PLoS One. 2015 Oct 9;10(10):e0140023. doi: 10.1371/journal.pone.0140023 (PMC4599856; doi:10.1371/journal.pone.0140023)
Supplement: S1 Fig — a, 403 on-farm trials conducted on soils with various inherent productivities in the major Chinese rice cropping systems from 2008–2011; b, Yield data in zero-N conditions derived from 5351 locations for assessment of inherent soil productivity of major rice farming systems. (DOC) [file pone.0140023.s001.doc]

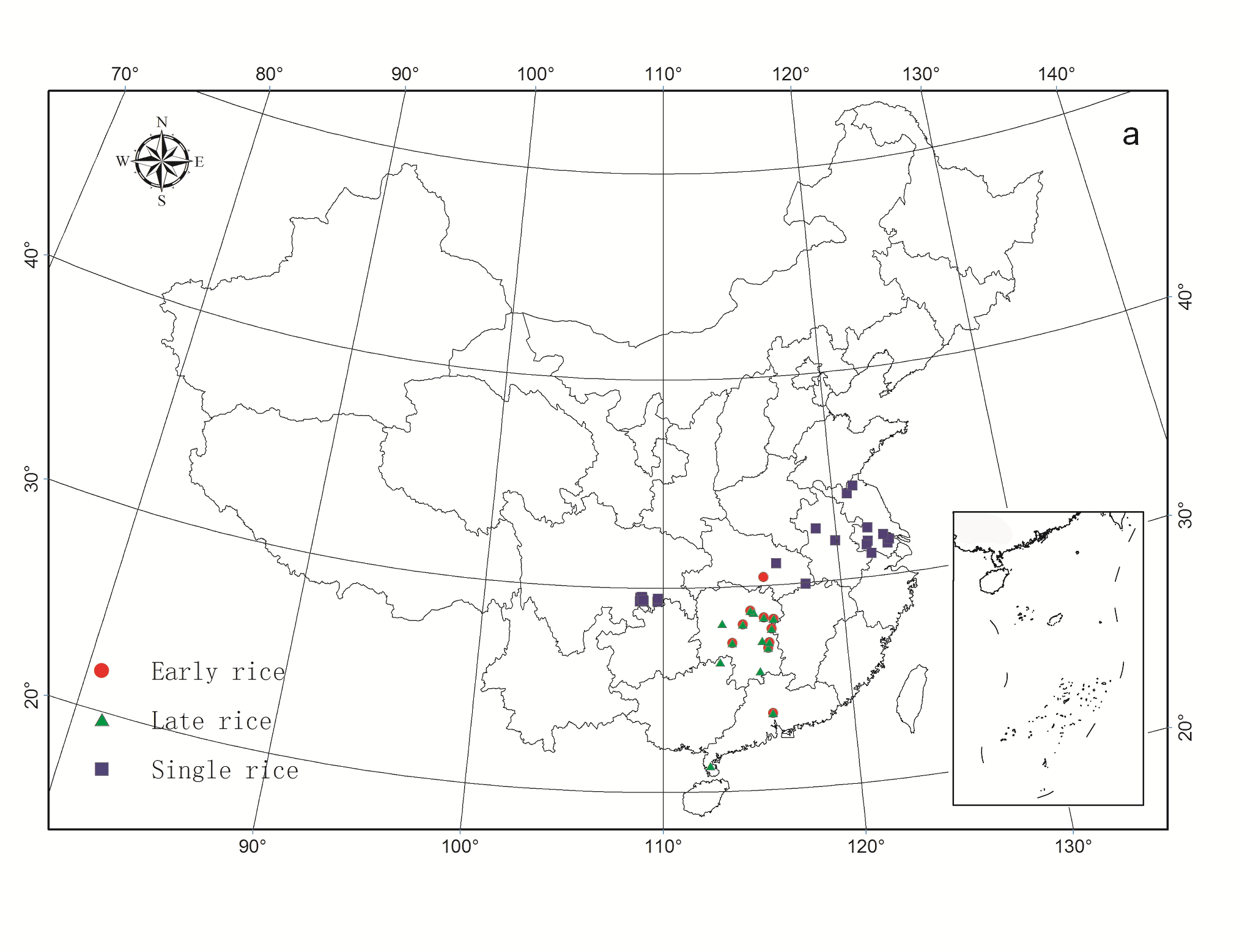


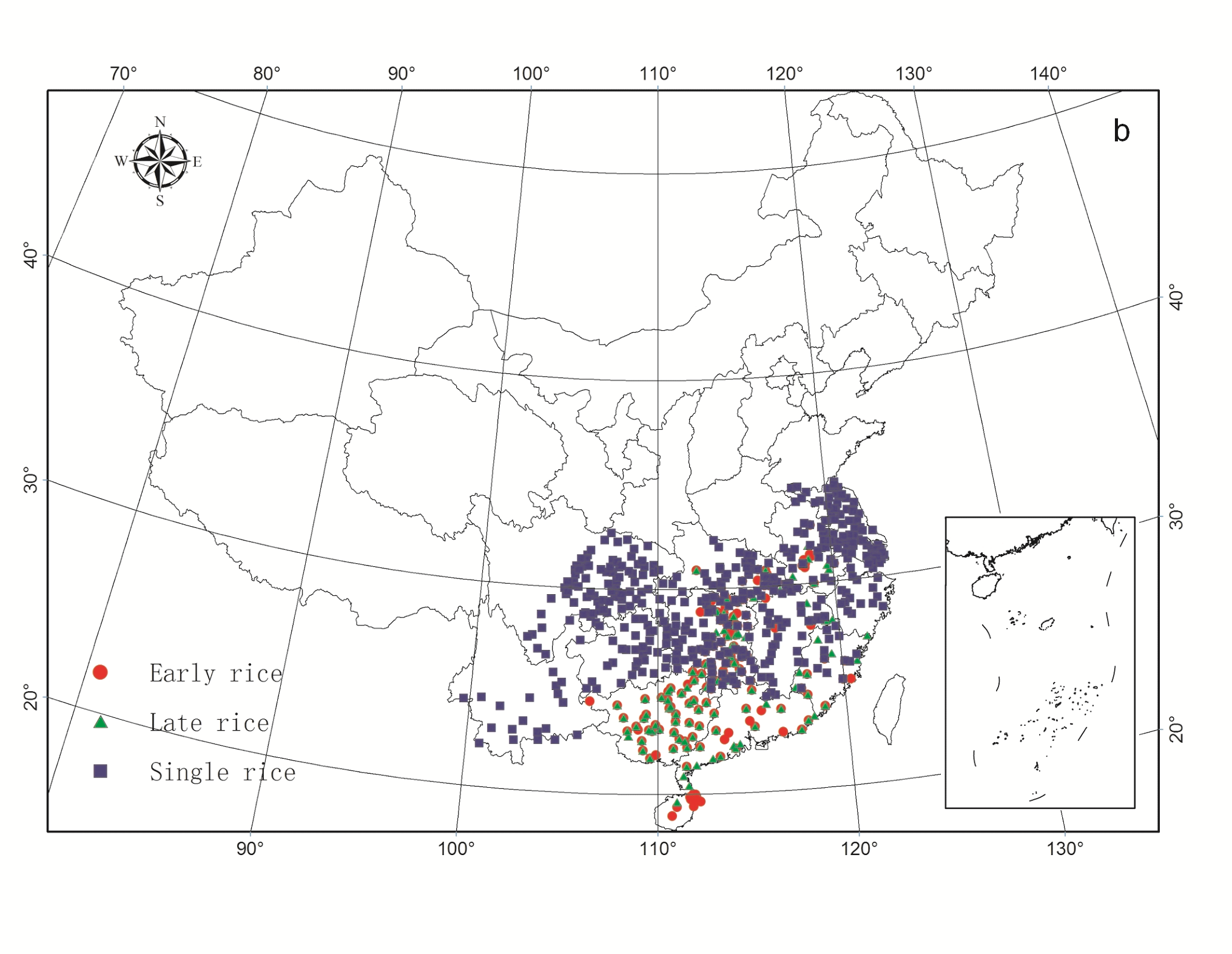


**S1 Fig. Geographical distribution of the dataset.** a, 403 on-farm trials conducted on soils with various inherent productivities in the major Chinese rice cropping systems from 2008-2011; b, Yield data in zero-N conditions derived from 5351 locations for assessment of inherent soil productivity of major rice farming systems. Literature sources and documents from which the data were derived are listed in Table C in the S1 Text.
