# Supplementary material for: Exploiting Co-Benefits of Increased Rice Production and Reduced Greenhouse Gas Emission through Optimized Crop and Soil Management
Source: PLoS One. 2015 Oct 9;10(10):e0140023. doi: 10.1371/journal.pone.0140023 (PMC4599856; doi:10.1371/journal.pone.0140023)
Supplement: S2 Fig — Black points represent F-D-F (n = 51) and white points represent F-D-F-M (n = 44). (DOC) [file pone.0140023.s002.doc]

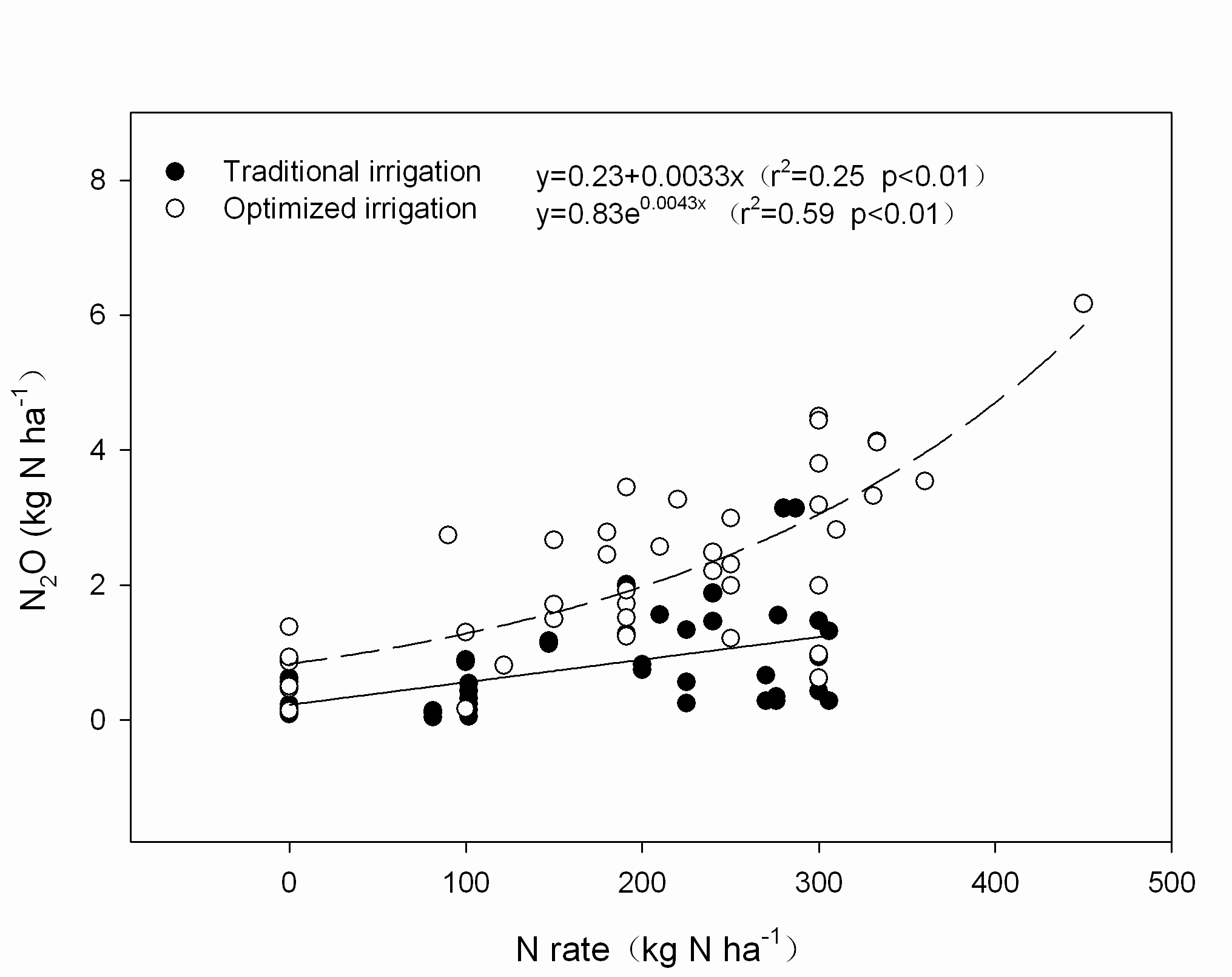


**S2 Fig. Relationships between nitrogen fertilizer rate and N_2_O emissions in paddy for traditional continuous flooding (F-D-F) and intermittent irrigation after midseason drainage (F-D-F-M) in south China and the Yangtze Delta.** Black points represent F-D-F (n=51) and white points represent F-D-F-M (n=44). The data and literature sources and documents from which the data were derived are listed in Table B in S1 Text.
