# Supplementary material for: Exploiting Co-Benefits of Increased Rice Production and Reduced Greenhouse Gas Emission through Optimized Crop and Soil Management
Source: PLoS One. 2015 Oct 9;10(10):e0140023. doi: 10.1371/journal.pone.0140023 (PMC4599856; doi:10.1371/journal.pone.0140023)
Supplement: S3 Fig — Dataset derived from 5351 locations. Early rice (n = 861), late rice (n = 1055), single rice (n = 3435) and rice (n = 5351). (DOC) [file pone.0140023.s003.doc]

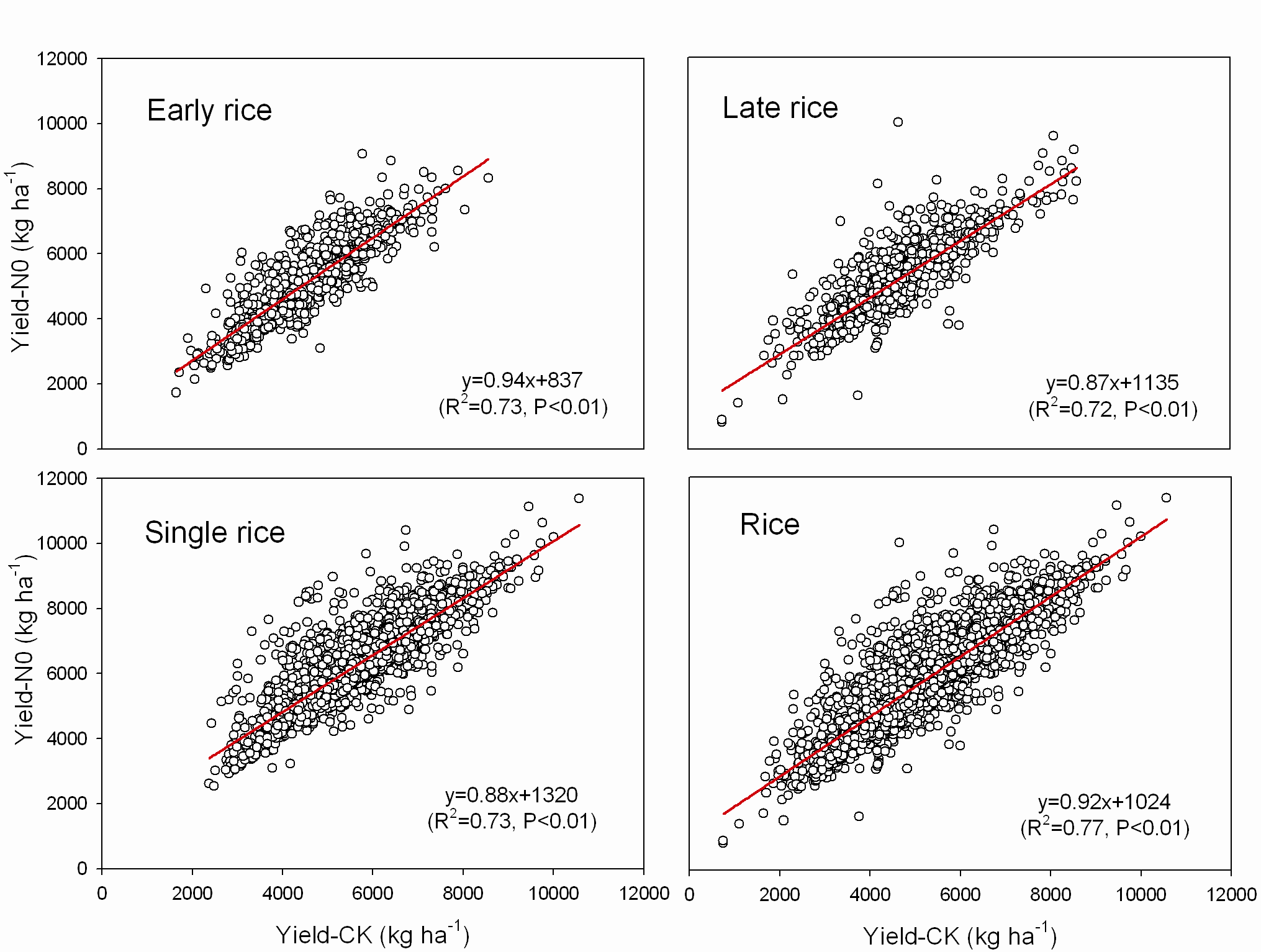


**S3 Fig. Relationships in rice yield between zero-N (Yield-N0) and fertilizer-omission plots (Yield-CK).** Dataset derived from 5351 locations and literature sources and documents from which the data were derived are listed in Table C in S1 Text. Early rice (n=861), late rice (n=1055), single rice (n=3435) and rice (n=5351).
