# Supplementary material for: Exploiting Co-Benefits of Increased Rice Production and Reduced Greenhouse Gas Emission through Optimized Crop and Soil Management
Source: PLoS One. 2015 Oct 9;10(10):e0140023. doi: 10.1371/journal.pone.0140023 (PMC4599856; doi:10.1371/journal.pone.0140023)
Supplement: S1 Table — (DOC) [file pone.0140023.s004.doc]

**S1 Table.** Total rice production, fertilizer N consumption and global warming potential (GWP) of N_2_O and CH_4_ following different strategies for early and late rice in south China and single rice in the Yangtze Delta.

| Scenario analysis* | Rice production (10^6^ Mg) | | | | | | Total N fertilizer consumption (10^6^ Mg) | | | | | | GWP (10^6^ Mg CO_2_-eq ) | | | | | |
| --- | --- | --- | --- | --- | --- | --- | --- | --- | --- | --- | --- | --- | --- | --- | --- | --- | --- | --- |
|  | Production | | | Increase‡ | | | N fertilizer consumption | | | Increase‡ | | | GWP | | | Increase‡ | | |
|  | Early | Late | Single | Early | Late | Single | Early | Late | Single | Early | Late | Single | Early | Late | Single | Early | Late | Single |
| FPs | 33.4†  (19.7%) | 37.2  (22.0%) | 98.7  (58.3%) | —— | —— | —— | 0.96†  (19.9%) | 1.10  (22.8%) | 2.76  (57.4%) | —— | —— | —— | 35.3†  (20.7%) | 86.4  (50.8%) | 48.6  (28.5%) | —— | —— | —— |
| Adopting BMPs | 36.3^§^  (19.5%) | 42.0  (22.5%) | 108.0  (58.0%) | 3.0^§^  (17.5%) | 4.7  (27.9%) | 9.2  (54.6%) | 0.77  (20.0%) | 0.88  (22.8%) | 2.20  (57.2%) | -0.19^§^  (19. 2%) | -0.22  (22.7%) | -0.56  (58.1%) | 32.4  (20.8%) | 76.2  (49.1%) | 46.8  (30.1%) | -2.9^§^  (19.6%) | -10.2  (68.5%) | -1.8  (11.9%) |
| Increasing ISP and FPs | 35.5  (19.2%) | 42.8  (23.1%) | 107.0  (57.7%) | 2.1  (13.3%) | 5.6  (34.9%) | 8.3  (51.8%) | 0.96  (19.9%) | 1.11  (23.0%) | 2.75  (57.1%) | 0  (-2.4%) | 0.02  (252.6%) | -0.01(-150.2%) | 36.1  (20.9%) | 87.4  (50.5%) | 49.3  (28.6%) | 0.8  (32.0%) | 0.9  (36.8%) | 0.8  (31.2%) |
| Adopting BMPs and Increasing ISP | 38.2  (19.1%) | 46.6  (23.3%) | 115.2  (57.6%) | 4.8  (15.7%) | 9.4  (30.6%) | 16.5  (53.7%) | 0.77  (20.5%) | 0.91  (24.2%) | 2.08  (55.3%) | -0.19  (17.7%) | -0.19  (17.6%) | -0.69  (64.7%) | 33.2  (21.0%) | 77.1  (48.9%) | 47.5  (30.1%) | -2.1  (16.9%) | -9.3  (74.9%) | -1.0  (8.2%) |

*Note*: *FPs, current farming practice continues; Adopting BMPs, adopting best management practices; Increase ISP and FPs, increasing inherent soil productivity by 1500 kg ha^-1^ for soils of low and moderate level but with adoption of current farming practice; and Adopting BMPs and Increasing ISP, a combination of adopting best management practices and increasing inherent soil productivity. † Data are absolute amount of rice production (10^6^ Mg), N fertilizer consumption (10^6^ Mg) and GWP (10^6^ Mg CO_2_-eq, as the sum of emission of CO_2_-eq of NO_2_ and CH_4_ during the rice growing season) under different scenario for each rice system and the relative contribution (%) to total of three rice systems. ‡ change in total rice production, N fertilizer consumption and GWP with adoption of scenario 2, 3 or 4 compared with FPs, and ^§^data are changes in absolute amounts for each rice system and relative contribution (%) to total changes of three rice systems
