# Supplementary material for: Exploiting Co-Benefits of Increased Rice Production and Reduced Greenhouse Gas Emission through Optimized Crop and Soil Management
Source: PLoS One. 2015 Oct 9;10(10):e0140023. doi: 10.1371/journal.pone.0140023 (PMC4599856; doi:10.1371/journal.pone.0140023)
Supplement: S1 Text — (DOC) [file pone.0140023.s005.doc]

**Supporting information for**

**Exploiting co-benefits of increased rice production and reduced greenhouse gas emission through optimized crop and soil management**

Ning An^1^, Mingsheng Fan^1*^, et al.,

*To whom correspondence should be addressed. Email: [fanms@cau.edu.cn](mailto:fanms@cau.edu.cn), Tele: 86-10-62731661; Fax: 86-10-62731016

**S1 Text Supplementary materials and methods**

**1. Classification of inherent soil productivity of major rice production systems**

The importance of soils to crop production is evident. However, quantifying the effects of soils on crop production by specific soil properties or a set of soil properties remains a challenge because soil properties interact with each other to impact on crop growth and yield with both trade-offs and compensating effects. Moreover, flooded rice fields behave very differently from non-flooded soils in many processes that affect the availability of nutrients or other soil functions. Consequently, many commonly available soil testing methods fail to properly predict soil nutrient supplying capacity under such conditions [1]. Thus, a plant-based approach has been proposed to serve as a bioassay of the interacting soil properties in the assessment of indigenous nutrient supply capacity and soil quality [2-4]. Previous studies also showed that N deficiency is a universal and key yield-limiting factor in irrigated rice systems [2,4]. Thus, the indigenous capacity of the soil to supply N (estimated as the actual yield of rice without N application but receive P and K (zero-N) was taken as the measurement of inherent soil productivity in Chinese rice farming systems in the current study. However, the viability of assessment was tested by comparison of yields between zero-N and fertilizer-omission plots using a national-scale dataset. Further, we checked the average SOC, total N concentration and pH between different soil grades of inherent productivity with an attempt to ascribe yield in zero-N plot to specific soil properties.

Due to locations of 403 on-farm trails were not selected randomly, yield data in N omission in these trails could not be used in assessing the current state of inherent soil productivity of rice cropping systems at the national scale. Thus, the national scales’ assessment of the current status of inherent productivity of paddy soils depended on yield data during 1-2 years in zero-N plots derived from 5351 trials conducted from 2000 to 2010 across the double rice region in the south and single rice systems in the Yangtze Delta. These trials also included the fertilizer-omission plots. A total of 861 trials were conducted on early rice, 1055 on late rice, and 3435 on single rice. A total of 177 published papers and documents from which the data were derived are listed in Table C. The geographical distribution of the sites is shown in S1 Fig.

Based on the principles of classification of arable land quality in China [5], paddy soils were graded according to yields of zero-N plots in on-farm trials as soils with < 3500, 3500-5000, and > 5000 kg ha^-1^ inherent productivity for early rice, < 4000, 4000-5500, and > 5500 kg ha^-1^ for late rice, and < 4500, 4500-6000, 6000-7500 and >7500 kg ha^-1^ for single rice.

The areas of the different soil grades of inherent productivity were calculated using the equation (Eq. S.1):

$\text{Area}_{\text{i}}=\text{d}_{\text{i}}\times\text{Area}_{\text{t}}\text{ }$ (S.1)

Where, Area_i_ represents the area of each grade of inherent soil productivity for early, late and single rice; i is the soil grade of inherent productivity; d_i_ represents the frequency distribution of different soil grades of inherent productivity for each of the three rice types; and Area_t_ is the total rice area for each rice type (5.8 × 10^6^ ha and 6.2 × 10^6^ ha for early and late rice in south China and 12.5 × 10^6^ ha for single rice in the Yangtze Delta) [6].

**2. Field-scale estimation of yield gap, N_2_O and CH_4_ emissions, global warming potential and greenhouse gas intensity**

The yield gap was estimated on the basis of datasets derived directly from 403 on-farm trials (Geographical distribution of the sites see S1 Fig.). The yield gap (Yield-Gap, kg ha^-1^) is the difference between yields under FP on soils with various inherent productivity and ‘attainable yields’ determined as mean yields of the 20% highest-yielding locations under BMPs. The details of FPs and BMPs are documented in Table A. In this approach, the attainable yield is a conservative estimate of landscape-scale achievable yield during 2008-2011 and not an estimate of physiologically achievable potential yield. Thus, yield gap represents the practical and exploitable yield gap and was estimated using the equation (Eq. S.2):

$\text{Yield-Gap}=\text{Yield}_{\text{a}}\text{-Yield}_{\text{f}}\text{ }$ (S.2)

Where, Yield_a_ is the attainable yield and Yield_f_ is the yield on a range of soil grades of inherent productivity under FPs in on-farm trials for the three rice farming systems.

N_2_O emissions (kg N ha^-1^) for both FPs and BMPs were calculated according to the N rate in each of the 403 on-farm trials using equations (Eq. S3 and S4) and on the basis of N_2_O emission–N input response curves of F-D-F and F-D-F-M (S2 Fig.):

$\text{N}_{2}\text{O-N emission }=\text{ a }+\text{ b}\times\text{N}_{\text{r}}\text{ }$ (S.3)

Where, N_r_ is N fertilizer rate in each on-farm trial for FPs; “a” and “b” represent the intercept and slope of the equations of N_2_O emission versus N rate for traditional flooding irrigation (S2 Fig.).

N_2_O-N emission = 0.83e ^0.0043Nr^  (S.4)

Where, N_r_ is fertilizer N rate in each on-farm trial for BMPs. Data for establishing S.3 and S.4 were derived from 95 observations in 35 fields in 29 published sources (Table B)

The CH_4_ emission was calculated based on soil inherent productivity grades at rice farming type scale by using modifying model [10], which relates CH_4_ ﬂux in the rice growing season to soil properties, water regime in the rice growing season, water status in the previous season, organic amendment and climate. The statistical model in the current study is expressed as equation (Eq. S.5) because of the absence of any organic amendment:

$\text{ln(flux)}=\text{constant}+\text{a}\times\text{ln(SOC)}+\text{ pH}_{\text{m}}\text{ }+\text{ PW}_{\text{i}}+\text{ WT}_{\text{j}}\text{ }+\text{ CL}_{\text{k}}\text{ }$ (S.5)

enehenS?arm trails. the existing000000000000000000000000000000000000000000000000000000000000000000000000000000000000000000000000Where, ﬂux (kg ha^-1^) is the average CH_4_ ﬂux during the rice-growing season; SOC is soil organic carbon concentration and “a” is the effective efficiency of SOC；pH_m_ is the effect of soil pH (m represents the pH classes); PW_i_ is the effect of pre-season water status (i is flooded, long drainage, short drainage or double drainage); WT_j_ is the effect of water regime in the rice-growing season (j is continuous flooding, single drainage or multiple drainage); and CL_k_ is the effect of climate. The water regime in the rice-growing season is classified as single drainage for FP (with one mid-season drainage and an end-of-season drainage) and multiple drainage for BMPs (with intermittent irrigation). The pre-season water regime is determined as permanently ﬂooded for late rice, long drainage for single rice in the Yangtze River Delta (where rice is rotated with upland crops) and short drainage for early rice (more than one month fallow). The mean SOC concentration and pH for each of the inherent soil productivity grades summarized in a national scale dataset (Table D) and on-farm trails were adopted for calculation of CH_4_ emission. The single rice system is practiced in the warm sub-humid subtropical zone with summer rainfall and the double rice system is practiced in the warm/cool humid subtropical zone with summer rainfall [11].

The global warming potential (GWP, kg CO_2_-eq ha^-1^) of N_2_O and CH_4_ emissions

$\text{GWP}=298\times\text{N}_{2}\text{O-N}\times\text{44/28}+\text{CH}_{4}\times\text{25 }$over a 100-year time horizon at the area scale from rice fields during the rice growing season was calculated using the equation (Eq. S.6):

(S.6)

Where, N_2_O-N is total N_2_O emissions (kg N ha^-1^) during the rice growing season and 44/28 is the conversion coefficient for N to N_2_O; 298 is the conversion coefficient for 1 kg N_2_O to equivalent CO_2_; where CH_4_ is total CH_4_ emissions (kg ha^-1^) during the rice growing season and 25 is the conversion coefficient for 1 kg CH_4_ to equivalent CO_2_ [12].

GHG intensity (GHG) reflects the GHG emissions per unit yield. It also considers associated GWP from clearing of carbon-rich natural ecosystems for crop production and recognizes the potential for indirect land use [13-15]. GHGI was calculated using the equation (Eq. S.7):

$\text{GHGI}=\text{net-GWP}\times10^{3}\text{/Y }$ (S.7)

Where GHGI represents GHG intensity (kg CO_2_-eq Mg^-1^ grain yield), GWP is global warming potential of N_2_O and CH_4_ (kg CO_2_-eq ha^-1^), and Y is rice yield (Mg ha^-1^)

Following the above data processing the linear regressions were conducted between yields in zero-N plots (Yield-N0, representing the inherent soil productivity) and Yield-BMPs, Yield-FPs, Yield gap, CH_4_ emissions, GWP and GHGI for the three rice systems based on 403 on-farm trials. The exponential regressions between yield-N0 and N_2_O emissions for both BMPs and FPs over three rice systems were summarized.

Factors tested were considered to be statistically significant at p < 0.05. All statistical analysis was performed using the SPSS software package (SPSS 13.0, SPSS Inc).

**3. Calculation of response of BMPs in terms of yield and greenhouse gas emissions**

The response of BMPs (Res-BMPs) is defined as the difference in agronomic performance and environmental impact between BMPs and FPs. The intent is to indicate to what extent BMPs can enhance rice productivity and reduce the environmental footprint. It was calculated for each soil grade of inherent productivity for the three rice systems by using equation (Eq.S.8):

$\text{Res-BMPs }=\text{ X-BMPs - X-FPs }$ (S.8)

Where, Res-BMPs was response of BMPs; X-BMPs and X-FPs represent rice yield, N fertilizer rate, emission of N_2_O and CH_4_, GWP and GHGI of CH_4_ and N_2_O under BMPs and FPs, respectively.

**4. Nationwide evaluation of total rice production, N fertilizer consumption and greenhouse gas emissions following alternative strategies**

Total national rice production, N fertilizer consumption and GHG emissions across the major rice cropping systems were evaluated for the four strategies: (1) current farming practices continue; (2) the BMPs currently available are adopted; (3) soils with low or moderate inherent productivity are improved but with continuation of current farming practices, and (4) a combination of adopting BMPs and increasing inherent soil productivity. For perspectives of increasing inherent soil productivity in strategies 3 and 4 we assumed that inherent soil productivity of >5000 kg ha^-1^ for early rice, >5500 kg ha^-1^ for late rice, and >7500 kg ha^-1^ for single rice were the potential levels, and soils with inherent productivity below these levels would be improved by 1500 kg ha^-1^. The latter is in accord with a national target in shifting current soils with low inherent productivity to moderate, and moderate to high levels during the coming decades [16].

Total rice production (10^6^ Mg), N_2_O (10^3^ Mg), CH_4_ (10^4^ Mg) emission and GWP (10^6^ Mg CO_2_-C-eq) across the major rice cropping systems were calculated according to the equation (Eq. S.9):

(S.9)

Where, TRP, TN_2_O, TCH_4_ and TGWP are total rice production, N_2_O and CH_4_ emissions and GWP across the major double rice system in south China and single rice in the Yangtze Delta following different strategies; i is soil grade with different inherent productivity; Y/N_2_O/CH_4_/GWP_i-e_, Y/N_2_O/CH_4_/GWP_i-l_ and Y/N_2_O/CH_4_/GWP_i-s_ represent the mean yield, N_2_O, CH_4_ emissions or GWP under each of the soil grades of different inherent productivity for early, late and single rice systems, which were calculated according to the model between yield, N_2_O, CH_4_ or GWP and inherent soil productivity for both FPs and BMPs (Fig. 1 and Fig. 4a-c); and Area_i-e_, Area_i-l_ and Area_i-s_ represent the areas of each soil grade with different inherent productivity for early, late and single rice systems, respectively (Table D). The area of each soil grade of inherent productivity for the three rice systems for strategies 1 and 2 was estimated according to equation (1), which represents the current actual area for each rice type. The areas of 3500-5000 kg ha^-1^ and 4000-5500 kg ha^-1^ grades for strategies 3 and 4 were areas of <3500 kg ha^-1^ and <4000 kg ha^-1^ grade which were otherwise without soil improvement for early and late rice, respectively; areas >5000 kg ha^-1^ and >5500 kg ha^-1^ are the sum of the current areas of 3500-5000 kg ha^-1^ and >5000 kg ha^-1^ for early rice and 4000-5500 kg ha^-1^ and >5500 kg ha^-1^ for late rice. For single rice systems the 4500-6000 kg ha^-1^ and 6000-7500 kg ha^-1^ grades were areas of <4500 kg ha^-1^ and 4500-6000kg ha^-1^ grade which were otherwise without soil inherent productivity improvement, respectively; and the areas >7500 kg ha^-1^ were the sum of the current areas of 6000-7500 kg ha^-1^ and >7500 kg ha^-1^ of inherent productivity.

Total fertilizer N consumption (TNFC, 10^6^ Mg) across the major rice cropping systems was calculated using the equation (Eq. S.10):

(S.10)

Where, TNFC is the total amount of fertilizer N applied across the major double rice systems in south China and the single rice systems in the Yangtze Delta following different strategies; NFC_i-e，_NFC_i-l_ and NFC_i-s_ are the exact mean fertilizer N rate for different soil inherent productivity grades for FPs or BMPs across 403 on-farm trials for the three rice farming systems, respectively; and i and Area_i_ are the same as in equation (S.9).

**Table A.** Detailed information on crop management for farming practice (FPs) and best management practice (BMPs) in 403 on-farm trials conducted in early and later rice systems in south China and single rice in the Yangtze Delta.

| Management | BMP | FP |
| --- | --- | --- |
| Nitrogen | - Integrated nutrient management (INM) principles [7, 8]. - Time and ratio: 3 or 4 times;   40-50% as basal, 20-30% tillering and 30% panicle fertilization for most locations. But about additional 5-10% of total rate would be applied based on whether conditions at rice grain filling stage.   - Mean applied rate (kg N ha^-1^):   Early, 132.8; Late, 142.3; and Single, 176.0. | - Time and ratio: 2 or 3 times;   60-80% as basal and 20-40% tillering fertilization for most of locations. Or 60% as basal, 20% tillering and 20% panicle fertilization for 47 locations.   - Mean applied rate (kg N ha^-1^):   Early, 164.9; Late, 177.3; Single, 227.4. |
| Phosphorus | - INM principles [7, 8]. - Time and ratio: 100% as basal   fertilization   - Mean applied rate (kg P ha^-1^):   Early, 77.8; Late: 80.0; Single: 34.2. | - Time and ratio: 100% as basal   fertilization   - Mean applied rate (kg P ha^-1^):   Early, 85.9; Late, 84.6; Single, 68.0. |
| Potassicum | - INM principles [7, 8]. - Time and ratio: 100% as basal   or 50% as basal and 50% as panicle fertilization.   - Mean applied rate (kg P ha^-1^):   Early, 77.8; Late, 80.0; Single, 34.2. | - Time and ratio: 100% as basal   fertilization   - Mean applied rate (kg P ha^-1^):   Early, 85.9; Late, 84.6; Single, 68.0. |
| Water | Intermittent (or alternate dry and wet irrigation) irrigation: characterized by flooding -- midseason drainage – reflooding - drying-wetting alternation during the following period (F-D-F-M) [9].  Usually, F-D-F-M can save irrigation water by 20-30% than traditional continuous flooding (F-D-F). | Traditional continuous flooding: characterized by flooding -- midseason drainage -- reflooding (F-D-F).  Water consumption: 1500-2000 mm for one season. |
| Residue | Straw removed | Straw removed |
| Rice Density | Transplanting by hand with mean density of 26.6 thousand seeding ha^-1^. | Transplanting by hand with mean density of 22.2 thousand seeding ha^-1^. |
| Tillage | Conventional tillage | Conventional tillage |
| Pest and Disease Control | Normal pest and disease control | Normal pest and disease control |
| Rotation | Double rice: Early and late rice are grown in the same field each year. Single rice: rotated with other upland crops e.g. wheat or oilseed rape. | |

**Table B. Complete data and sources on fertilizer N rate, N_2_O emissions and water management regimes in early and late rice in south China and single rice in the Yangtze Delta.**

| No. | Yield-N0  (kgha^-1^) | N rate  (kg N ha^-1^) | 000000000000000000000000000000000000000000000000000000000000000000000000000000000000000000000000000000000000000000000000000000N_2_O emission( kg N ha^-1^) | Water  management | Language* | Type† | Author list | Journal, Dissertation, Monograph | Year Published, Volume and Pages |
| --- | --- | --- | --- | --- | --- | --- | --- | --- | --- |
| 1 | —— | 191 | 2.0 | F-D-F | C | [J] | Zheng XH, Wang MX, Wang YS, Shen RX, Gong YB, Zhang W, Luo DM. | Chinese Journal of Applied Ecology. | 1997; 8: 495-499. |
|  | —— | 191 | 2.0 | F-D-F |  |  |  |  |  |
|  | —— | 191 | 1.3 | F-D-F |  |  |  |  |  |
| 2 | —— | 102 | 0.4 | F-D-F | C | [D] | Wu PP. | Nanjing Agricultural University. | 2005. |
|  | —— | 102 | 0.5 | F-D-F |  |  |  |  |  |
|  | —— | 102 | 0.1 | F-D-F |  |  |  |  |  |
|  | —— | 82 | 0.1 | F-D-F |  |  |  |  |  |
|  | —— | 82 | 0.1 | F-D-F |  |  |  |  |  |
|  | —— | 82 | 0.0 | F-D-F |  |  |  |  |  |
|  | —— | 102 | 0.2 | F-D-F |  |  |  |  |  |
|  | —— | 102 | 0.3 | F-D-F |  |  |  |  |  |
|  | —— | 102 | 0.1 | F-D-F |  |  |  |  |  |
| 3 | —— | 0 | 0.6 | F-D-F | C | [J] | Liang GQ, Zhou W, Xia WJ, Wang XB, Sun JW. | Plant Nutrition and Fertilizer Science. | 2010; 16: 304-311. |
|  | —— | 210 | 1.6 | F-D-F |  |  |  |  |  |
|  | —— | 147 | 1.2 | F-D-F |  |  |  |  |  |
|  | —— | 147 | 1.1 | F-D-F |  |  |  |  |  |
| 4 | —— | 225 | 1.3 | F-D-F | C | [J] | Li ML, Xu YC, Shen QR, Zhou CL, Huang XY, Yin XY, Yin JL, Klaus D. | Acta Pedologica Sinica. | 2003; 40: 864-869. |
| 5 | 5620 | 0 | 0.1 | F-D-F | E | [J] | Ma YC, Kong XW, Yang B, Zhang XL, Yan XY, Yang JC, Xiong ZQ. | Agriculture, Ecosystems and Environment. | 2013; 164: 209-219. |
|  | 5620 | 300 | 0.4 | F-D-F |  |  |  |  |  |
|  | 5620 | 225 | 0.3 | F-D-F |  |  |  |  |  |
|  | 5620 | 270 | 0.3 | F-D-F |  |  |  |  |  |
|  | 5910 | 0 | 0.5 | F-D-F |  |  |  |  |  |
|  | 5910 | 300 | 0.6 | F-D-F |  |  |  |  |  |
|  | 5910 | 225 | 0.6 | F-D-F |  |  |  |  |  |
|  | 5910 | 270 | 0.7 | F-D-F |  |  |  |  |  |
| 6 | —— | 100 | 0.9 | F-D-F | E | [J] | Qin YM, Liu SW, Guo YQ, Liu QH, Zou JW. | Biology and Fertility of Soils. | 2010; 46: 825-834. |
|  | —— | 100 | 1.3 | F-D-F-M |  |  |  |  |  |
| 7 | —— | 0 | 0.2 | F-D-F | E | [J] | Xiong ZQ, Xing GX, Tsuruta H, Shen GY, Shi SL, Du LJ. | Nutrient Cycling in Agroecosystems. | 2002; 64: 125-133. |
|  | —— | 276 | 0.4 | F-D-F |  |  |  |  |  |
|  | —— | 0 | 0.2 | F-D-F |  |  |  |  |  |
|  | —— | 276 | 0.3 | F-D-F |  |  |  |  |  |
| 8 | —— | 0 | 0.1 | F-D-F | E | [J] | Cai ZC, Xing GX, Yan XY, Xu H, Tsuruta H, Yagi K, Minami K. | Plant and Soil | 1997; 196: 7–14. |
|  | —— | 100 | 0.2 | F-D-F |  |  |  |  |  |
|  | —— | 100 | 0.2 | F-D-F |  |  |  |  |  |
|  | —— | 300 | 0.6 | F-D-F |  |  |  |  |  |
|  | —— | 300 | 1.0 | F-D-F |  |  |  |  |  |
| 9 | —— | 277 | 1.6 | F-D-F | E | [J] | Zou JW, Huang Y, Jiang JY. | Global Biogeochemical Cycles | 2005; 19: GB2021. |
|  | —— | 333 | 4.1 | F-D-F-M |  |  |  |  |  |
| 10 | —— | 0 | 0.6 | F-D-F | C | [J] | Cao JL, Xu H, Zhang HK, Xing GX, Ren LT, Yang BL. | Chinese Journal of Ecology. | 1999; 18: 6-9. |
|  | —— | 100 | 0.9 | F-D-F |  |  |  |  |  |
|  | —— | 200 | 0.8 | F-D-F |  |  |  |  |  |
|  | —— | 200 | 0.7 | F-D-F |  |  |  |  |  |
|  | —— | 300 | 0.9 | F-D-F |  |  |  |  |  |
| 11 | —— | 287 | 3.1 | F-D-F | C | [J] | Yang H, Wu LP, Yang C, Chen YF, Lv XJ, Wu SZ. | Journal of South China Agriculture University. | 1997; 18: 62-66. |
| 12 | —— | 280 | 3.1 | F-D-F | C | [J] | Yang J, Chen YF, Hu F, Wu SZ, Wang GC. | Journal of South China Agriculture University. | 1996; 17: 52–57. |
| 13 | —— | 306 | 0.3 | F-D-F | C | [J] | Lu WS, Zhang JG, Liao CW. | Chinese Journal of Applied Ecology. | 1997; 8: 275–278. |
|  | —— | 306 | 1.3 | F-D-F |  |  |  |  |  |
| 14 | 8380 | 0 | 0.1 | F-D-F | E | [J] | Liu XY, Qu JJ, Li LQ, Zhang AF, Zheng JF, Zheng JW, Pan GX. | Ecological Engineering. | 2012; 42: 168-173. |
|  | 8380 | 240 | 1.9 | F-D-F |  |  |  |  |  |
|  | 6380 | 0 | 0.2 | F-D-F |  |  |  |  |  |
|  | 6380 | 240 | 1.5 | F-D-F |  |  |  |  |  |
|  | 6670 | 0 | 0.1 | F-D-F |  |  |  |  |  |
|  | 6670 | 300 | 1.5 | F-D-F |  |  |  |  |  |
| 15 | —— | 122 | 0.8 | F-D-F-M | C | [J] | Yuan WL, Cao CG, Cheng JP, Xie NN. | Scientia Agricultura Sinica. | 2008; 41: 4294-4300. |
| 16 | —— | 300 | 2.0 | F-D-F-M | E | [J] | Zhang AF, Bian RJ, Pan GX, Cui LQ, Hussaina Q, Li LQ, Zheng JW, Zheng JF, Zhang XH, Han XJ, Yu XY. | Field Crops Research. | 2012; 127: 153-160. |
|  | —— | 300 | 4.5 | F-D-F-M |  |  |  |  |  |
| 17 | —— | 240 | 2.5 | F-D-F-M | C | [J] | Zhang YF, Chen LG, Wang ZC, Zhang CS, Zhu PP, Sheng J, Zheng JC. | Journal of Agro-Environment Science. | 2010; 29: 1403-1409. |
|  | —— | 240 | 2.2 | F-D-F-M |  |  |  |  |  |
| 18 | —— | 240 | 2.2 | F-D-F-M | C | [J] | Zhang YF, Zheng,JC, Chen LG, Wang ZC, Zhu PP, Shen J, Wang YL. | Ecology and Environmental Sciences. | 2009; 8: 2334-2338. |
| 19 | —— | 333 | 4.1 | F-D-F-M | C | [J] | Zou JW, Huang Y, Zong LG, Wang YS, Ronald L. S. | Environmental Science. | 2003; 24: 7-12. |
| 20 | —— | 300 | 3.2 | F-D-F-M | C | [J] | Chen ST, Huang Y, Zheng XH, Chen YQ. | Climatic and Environmental Research. | 2007; 12: 147-154. |
| 21 | 5203 | 0 | 0.1 | F-D-F-M | E | [J] | Cai ZC, Xing GX, Yan XY, Xu H, Tsuruta H, Yagi K, Minami K. | Plant Soil. | 1997; 196: 7-14. |
|  | 5203 | 100 | 0.2 | F-D-F-M |  |  |  |  |  |
|  | 5203 | 100 | 0.2 | F-D-F-M |  |  |  |  |  |
|  | 5203 | 300 | 0.6 | F-D-F-M |  |  |  |  |  |
|  | 5203 | 300 | 1.0 | F-D-F-M |  |  |  |  |  |
| 22 | —— | 0 | 1.4 | F-D-F-M | E | [J] | Zou JW, Huang Y, Lu YY, Zheng XH, Wang YS. | Atmospheric Environment. | 2005; 39: 4755-4765. |
|  | —— | 150 | 2.7 | F-D-F-M |  |  |  |  |  |
|  | —— | 300 | 4.4 | F-D-F-M |  |  |  |  |  |
|  | —— | 450 | 6.2 | F-D-F-M |  |  |  |  |  |
| 23 | —— | 150 | 1.5 | F-D-F-M | E | [J] | Zheng XH, Han SH, Huang Y, Wang YS, Wang MX. | Global Biogeochemical Cycles. | 2004; 18: GB2018. |
|  | —— | 250 | 2.3 | F-D-F-M |  |  |  |  |  |
|  | —— | 250 | 1.2 | F-D-F-M |  |  |  |  |  |
|  | —— | 0 | 0.9 | F-D-F-M |  |  |  |  |  |
|  | —— | 150 | 1.7 | F-D-F-M |  |  |  |  |  |
|  | —— | 250 | 2.0 | F-D-F-M |  |  |  |  |  |
|  | —— | 250 | 3.0 | F-D-F-M |  |  |  |  |  |
| 24 | —— | 0 | 0.9 | F-D-F-M | E | [J] | Xing GX, Zhu ZL. | Nutrient Cycling in Agroecosystems. | 1997; 49: 17-22. |
|  | —— | 210 | 2.6 | F-D-F-M |  |  |  |  |  |
|  | —— | 220 | 3.3 | F-D-F-M |  |  |  |  |  |
|  | —— | 310 | 2.8 | F-D-F-M |  |  |  |  |  |
| 25 | —— | 0 | 0.5 | F-D-F-M | E | [J] | Zheng XH, Wang MX, Wang YS, Shen RX, Gou J, Li J, Jin JS, Li LT. | Chemosphere Global Change Science. | 2000; 2: 207-224. |
|  | —— | 191 | 1.2 | F-D-F-M |  |  |  |  |  |
|  | —— | 191 | 1.7 | F-D-F-M |  |  |  |  |  |
|  | —— | 191 | 1.5 | F-D-F-M |  |  |  |  |  |
|  | —— | 0 | 0.5 | F-D-F-M |  |  |  |  |  |
|  | —— | 191 | 3.5 | F-D-F-M |  |  |  |  |  |
|  | —— | 191 | 1.9 | F-D-F-M |  |  |  |  |  |
| 26 | —— | 0 | 0.9 | F-D-F-M | C | [J] | Liu H, Zhao P, Lin YB, Rao XQ. | Journal of Tropical and Subtropical Botany. | 2006; 14: 269-274. |
|  | —— | 180 | 2.5 | F-D-F-M |  |  |  |  |  |
| 27 | —— | 331 | 3.3 | F-D-F-M | C | [J] | Jiang CS, Wang YS, Zheng XH, Zhu B, Huang Y. | Environmental Science. | 2006; 27: 207-213. |
| 28 | —— | 90 | 2.7 | F-D-F-M | C | [J] | Wen W, Lu J, Cao JM. | China Environmental Science. | 2005; 25: 540-543. |
|  | —— | 180 | 2.8 | F-D-F-M |  |  |  |  |  |
|  | —— | 360 | 3.5 | F-D-F-M |  |  |  |  |  |
| 29 | —— | 300 | 3.8 | F-D-F-M | C | [J] | Chen ST, Huang Y, Zheng XH, Chen YQ. | Scientia Agricultura Sinica. | 2005; 38: 2053-2060. |

*Note*: * C and E denote Chinese and English, respectively; † J, D, M denote Journal (articles), Dissertations, Monographs, respectively; F-D-F, Traditional continuous flooding characterized by flooding-midseason drainage-reflooding; F-D-F-M, Intermittent irrigation characterized by flooding - midseason drainage -reflooding- moist intermittent irrigation, but without water logging.

**Table C.** Complete data source list for assessment of inherent soil productivity of major rice farming systems.

| No. | Language* | Type† | Author list | Journal, Dissertation, Monograph | Year Published,  Volume and Pages |
| --- | --- | --- | --- | --- | --- |
| 1 | C | [J] | Zou YB, Tang QY, Huang JL, Shi JC, Zao XZ, Wang SB. | Review of China Agricultural Science and Technology. | 2003; 5: 36-41. |
| 2 | C | [J] | Zhou YB. | China Agricultural Technology Extension. | 2006; 22: 35-37. |
| 3 | C | [J] | Yu CL, Feng ZF. | Zhejiang Agricultural Sciences. | 2007; 4: 442-443. |
| 4 | C | [J] | Wang F, Luo T. | Fujian Rice Technology. | 2002; 20: 18-19. |
| 5 | C | [J] | Dong SQ. | Journal of Agricultural. | 2006; 12: 60-62. |
| 6 | C | [J] | Zeng LZ, Zeng HW, Zeng Z, Luo WL, Luo LH, Xu JH, Huang ZJ, Chen ZQ. | Guangdong Agricultural Sciences. | 2009; 4: 60-61. |
| 7 | C | [J] | Zhou B. | Journal of Hebei Agricultural Sciences. | 2007; 11: 14, 20. |
| 8 | C | [J] | Wang AP, Deng JL. | Crops. | 2006; 5: 28-30. |
| 9 | C | [J] | Zheng LJ, Zeng GY, Wang PF. | Chinese Agricultural Science Bulletin. | 2001; 17: 48-50. |
| 10 | C | [J] | Wang RF, Huang DM, Chui Y. | Soil and fertilizer. | 2002; 2: 3-8. |
| 11 | C | [M] | Zhu ZL, Zhang FS. (eds) | Basic Research on Nitrogen Behavior and Efficient Utilization in Major Agoecosystems in China (Science press, Beijing) | 2009. pp. 232. |
| 12 | C | [J] | Liu AY. | Journal of Shanghai Agricultural Science and Technology. | 2008; 5: 46-47. |
| 13 | C | [J] | Xie JX, Wu JS, Tan HF, Wang JD, Zhang YC. | Jiangsu Agricultural Sciences. | 2008; 5: 258-260. |
| 14 | C | [J] | Zhang G. | Journal of Anhui Agricultural Sciences. | 2005; 33: 2277-2278. |
| 15 | C | [J] | Tao YF. | Journal of Shanghai Agricultural Science and Technology. | 2003; 4: 97-98. |
| 16 | C | [J] | Wang YQ, Guo XS, W J. | Journal of Anhui Agricultural Science. | 2009; 37: 4923-4924. |
| 17 | C | [J] | Zhang XM, Guo XS. | Reclaiming and Rice Cultivation. | 2007; 3: 48-51. |
| 18 | C | [J] | Wang SF, Chen HL, Liu H. | Tillage and Cultivation. | 2002; 3: 38-30. |
| 19 | C | [J] | Ye HB, Huang XP, Yao M, Yin Y. | Journal of Shanghai Agricultural Science and Technology. | 2003; 3: 38-39. |
| 20 | C | [J] | Fei H, Dong YJ. | Rural Economy and Science Technology. | 2006; 10: 83-84. |
| 21 | C | [J] | Shao Y, Lu XW, Cong WY. | Shanghai Agricultural Science and Technology. | 2007; 3: 41, 42. |
| 22 | C | [J] | Wang YF, Mei GF, Ding B, Zhang Q, Zhu MP. | Barley and Cereal Sciences. | 2007; 4: 46-47, 48 |
| 23 | C | [J] | Qian FY, Zhu G, Zhu CM. | Modern Agriculture Science and Technology. | 2007; 21: 122. |
| 24 | C | [J] | Zhao SQ, Chen Q, Li DW. | Modern Agriculture Science and Technology. | 2007; 11: 100-104. |
| 25 | C | [J] | Shen YQ. | Agricultural Extension Services. | 2007; 24: 34. |
| 26 | C | [J] | Liu ZR, Rao HZ. | Tillage and Cultivation. | 2007; 3: 29, 58. |
| 27 | C | [J] | Li SQ, Chen JD, Zuo Q. | Journal of Anhui Agriculture Sciences. | 2007; 35: 8571-8573. |
| 28 | C | [J] | Wang SY, Kang ZM. | Modern Agriculture Science and Technology. | 2008; 5: 139. |
| 29 | C | [J] | Wang ZY, Gong CH, Yuan JH, Zhu DM, Zhou CL, Zhang HY, Zhou P. | Modern Agriculture Science and Technology. | 2008; 1: 111-113. |
| 30 | C | [J] | Sun GY, Fen PL, Gong CH, Yuan JH, Zhu DM, Zhang HY, Zhou CL. | Modern Agriculture Science and Technology. | 2008; 1: 106-108. |
| 31 | C | [J] | Li F, Liu LM, Bo TM, Zhao QM. | Anhui Agriculture Science Bulletin. | 2008; 14: 79, 133. |
| 32 | C | [J] | Yu ZJ, Tang LM, Chai LW. | Modern Agricultural Sciences and technology. | 2008; 6: 133-134. |
| 33 | C | [J] | Cheng JH, Zhang CE, Zheng L. | Journal of Hebei Agricultural Sciences. | 2009; 13: 56-59. |
| 34 | C | [J] | Tang DF, Jia HJ, Wang YF, Zeng CP, Li HA. | Modern Agriculture Science and Technology. | 2009; 3: 160-162. |
| 35 | C | [J] | Qian HJ. | Journal of South China Normal University (Natural Science Edition). | 2009; 1: 105-110. |
| 36 | C | [J] | Wu ZF. | Agricultural Extension Services. | 2007; 24: 41. |
| 37 | C | [J] | Fang XL. | Anhui Agriculture Science Bulletin. | 2007; 13: 46, 70. |
| 38 | C | [J] | Fang CS, Hu AJ. | Modern Agriculture Science and Technology. | 2007; 13: 137-138. |
| 39 | C | [J] | Zhu LF, Yu SM, Yang YN, Kang WQ, Dong CQ, Xu DH, Jin QY. | Chinese Journal of Rice Science. | 2007; 21: 631-636. |
| 40 | C | [J] | Liao TJ, Zhao JY, Huang Y, Rao ZH. | Journal of Southwest Agricultural University. | 2002; 24: 165-168. |
| 41 | C | [J] | Ma XY, Rui GH, Ye BL, Xu AQ. | Journal of Anhui Agricultural Sciences. | 2002; 30: 416, 439. |
| 42 | C | [J] | Ding GX, XuX, QianY, Zhao WP. | Journal of Anhui Agricultural Sciences. | 2006; 4: 115, 159. |
| 43 | C | [J] | Yang LH, Gu GM, Ni ZY, Pan YJ. | Journal of Shanghai Agricultural Science and Technology. | 2006; 5: 85. |
| 44 | C | [J] | Zhu ZL, Zhang SL, Yin B, Yan XY. | Plant Nutrition and Fertilizer Science. | 2010; 16: 1-5. |
| 45 | C | [J] | Lin YP, Cai HC. | Fujian Science & Technology of Tropical Crops. | 2002; 27: 6-8. |
| 46 | C | [J] | Mao XY, Liao ZW, Du JJ, Lin XJ. | Journal of South China Agricultural University (Natural Science Edition). | 2002; 23: |
| 47 | C | [J] | Chen ZQ, Li XH, Chen FS, Pan XF, Chen XY, Hu RJ, Wu J. | Anhui Agriculture Science Bulletin. | 2009; 15: 87-89, 125. |
| 48 | C | [J] | Zhang XM, Chen SL, Guo XS, Liu CS, Ye BC, Hu R, Ding XW, Tao CZ. | Journal of Anhui Agriculture Science. | 2006; 34: 4355-4356. |
| 49 | C | [J] | Fan DY, Mo SF, Jiang MY, Mo YJ, Chen HY. | Genomics and Applied Biology. | 2007; 26: 312-316. |
| 50 | C | [J] | Peng YX, Wang KR, Xie XL. | Soil and Fertilizer Sciences in China. | 2007; 4: 40-43, 48. |
| 51 | C | [J] | Zhou JM, Qian XM. | Journal of Zhejiang Agricultural Sciences. | 2007; 4: 436-438. |
| 52 | C | [J] | Zhang XM, Guo XS, Li ZF, Chen ZQ, Xia JF, Wang YL. | Chinese Journal of Soil Science. | 2008; 39: 1061-1066. |
| 53 | C | [J] | Zhang SH, Wu WG, Li ZF, Wang YL, Huang YD, Zhao JJ, Fang WJ. | Soil and Fertilizer Sciences in China. | 2008; 3: 28-31. |
| 54 | C | [J] | Wu WG, Yang LS, Zhao JJ, Hu GS, Fang WJ, Bai YS, Zhang SH, Zhang YH. | Journal of Anhui Agricultural University. | 2008; 35: 49-55. |
| 55 | C | [J] | Zhan QH, Chen J, Tan MZ, Sun ZY, Wang SQ. | Chinese Journal of Soil Science. | 2008; 39: 593-596. |
| 56 | C | [J] | Li JJ, Li H, Tang HL, Yue YJ. | Anhui Agriculture. | 2009; 4: 157. |
| 57 | C | [J] | Wu GR. | Fujian Science and Technology of Rice and Wheat. | 2001; 19: 21, 64. |
| 58 | C | [J] | Song ZZ, Xu JC. | Tillage and Cultivation. | 2002; 1: 33-34. |
| 59 | C | [J] | Huo ZY, Zhang HC, Wang XQ, Dai QG, Xu K, Ye QB. | Jiangsu Journal of Agricultural Sciences. | 2003; 19: 223-227. |
| 60 | C | [J] | Liu LJ, Sang DZ, Liu CL, Wang ZQ, Yang JC, Zhu QS. | Scientia Agricultura Sinica. | 2003; 36: 1456-1461. |
| 61 | C | [J] | Wang XQ, Zhang HC, Xu XJ. | Journal of South China Agricultural University (Natural Science Edition). | 2004; 25: 9-13. |
| 62 | C | [J] | Liu J, Wei YF, Xu SA. | Journal of Tianjin Agricultural College. | 2005; 12: 26-30. |
| 63 | C | [J] | Ding T, Zhang HC, Yuan QY. | Jiangsu Agricultural Sciences. | 2005; 1: 23-27. |
| 64 | C | [J] | Yao H, Zhang WM, Lu ZL. | Acta Agriculturae Jiangxi. | 2006; 18: 23-25. |
| 65 | C | [J] | Liu YY, Zhang HC, Hu X, Zhu DJ, Dai QG. | Soil and Fertilizer Sciences in China. | 2006; 4: 35-36. |
| 66 | C | [J] | Wang P, Liu H, Chen XL. | Tillage and Cultivation. | 2006; 5: 33-35. |
| 67 | C | [J] | Chen ZZ, Xu KH, Yu QE. | Journal of Hebei Agricultural Sciences. | 2007; 11: 69-70. |
| 68 | C | [M] | Wu CF, Liu LJ, Wang ZQ, Yang JC. | The professional committee of Chinese crop cultivation transition and academic seminars. | 2007. pp. 166-170. |
| 69 | C | [J] | Wang X, Xu CJ, Zhang CS, | Chinese Agriculture Science Bulletin. | 2007; 8: 77-78. |
| 70 | C | [J] | Wang HH, Shen MX, Liu FJ, Wu TD, Yao YM, Shen CY, Shen XP. | Jiangsu Agricultural Sciences. | 2007; 7: 9-11, 42. |
| 71 | C | [J] | Zhang L, Yao GQ, Wang BL, | Anhui Agriculture Science Bulletin. | 2007; 13: 112. |
| 72 | C | [J] | Yang XT, Lin XQ, Zend XY, Wang XH, Luo SZ. | Chinese Journal of Soil Science. | 2007; 38: 463-466. |
| 73 | C | [J] | Geng J. | Anhui Agriculture. | 2008; 7: 113, 115. |
| 74 | C | [M] | Chen ZM, Yan TW, Gu XL, Wang SJ. | Proceedings of Jiangsu cultivated land quality construction. | 2008. pp. 283-286. |
| 75 | C | [J] | Zhang YQ, Zhang Q, Mei CY, Wang MX. | Anhui Agriculture. | 2008; 13: 186-187. |
| 76 | C | [J] | Liu Y, Wang L, Wang CY. | Anhui Agriculture. | 2008; 15: 220-221. |
| 77 | C | [J] | Wang R, Cheng LL, Xiao GY. | Agricultural Technology Service. | 2008; 25: 24. |
| 78 | C | [J] | Yin CY, Wei HY, Zhang Q, Dai QG, Huo ZY, Xu K, Zhang SF, Hang J, Ma Q. | Acta Agronomica Sinica. | 2009; 35: 348-355. |
| 79 | C | [J] | Wu ZH, Qing XG, Yuan LP. | Journal of Hunan Agricultural University (Natural Sciences). | 2008; 34: 713-718. |
| 80 | C | [J] | Shi JP, Lu RK, Shi ZY, Sun B. | Acta Pedologica Sinica. | 2002; 39: 853-862. |
| 81 | C | [J] | Zhang QC, Wang GH. | Chinese Journal of Rice Sciences. | 2002; 16: 346-350. |
| 82 | C | [J] | Zhang XM, Guo XS, Liu CS, Hu R, Chen SL, Ye CB. | Journal of Anhui Agriculture Science. | 2005; 33: 1812-1813. |
| 83 | C | [J] | Zhang XM, Hu R, Chen SL, Ye CB, Guo XS, Liu CS. | Journal of Anhui Agricultural. | 2005; 33: 2021-2022, 2025. |
| 84 | C | [J] | Dai PA, Zheng SX, Yuan DR. | Hunan Agricultural Sciences. | 2002; 5: 21-24. |
| 85 | C | [J] | Zou YB, Ao HJ, Xia B, Tang QY, Peng SB, Rolang JB. | Crop Research. | 2008; 22: 214-219. |
| 86 | C | [J] | Wang SH, Cao WX, Ding YF, Liu SH, Wang QS. | Journal of Nanjing Agricultural University. | 2003; 26: 1-4. |
| 87 | C | [J] | Ding YF, Liu SH, Wang SH, Wang QS, Huang PS, Ling QH. | Acta Agronomica Sinica. | 2004; 30: 739-744. |
| 88 | C | [J] | Ye QB, Zhang HC, Wei HY, Zhang Y, Wang BF, Xia K, Huo ZY, Dai QG, Xu K. | Acta Agronomica Sinica. | 2005; 31: 1422-1428. |
| 89 | C | [J] | Yuan JC, Liu CJ, E SZ, Yang SM, Zhu QS, Yang JC. | Plant Nutrition and Fertilizer Science. | 2006; 12: 183-187. |
| 90 | C | [J] | Liu YY, Zhang HC, Song H, Dai QG, Huo ZY, Xu K. | Journal of Zhejiang Agricultural Sciences. | 2006; 3: 300-302. |
| 91 | C | [J] | Liu LJ, Xu W, Sang DZ, Liu CL, Zhou JL, Yang JC. | Acta Agronomica Sinica. | 2006; 32: 987-994. |
| 92 | C | [J] | Sun T, Xia LH. | Modern Agriculture Science and Technology. | 2007; 19: 128,130. |
| 93 | C | [M] | Qian HB, Zhu DJ, Liu YY, Yang JK, Wu TS, Yang H, Miu C. | Proceedings of Jiangsu cultivated land quality construction. | 2008. pp. 248-251. |
| 94 | C | [J] | Dai PA, Nie J, Zheng SX, Xiao J. | Chinese Journal of Soil Science. | 2003; 34: 115-119. |
| 95 | C | [J] | Wang DJ, Lin JH, Sun RJ, Xia LZ, Lian G. | Acta Pedologica Sinica | 2003; 40: 426-432 |
| 96 | C | [J] | Sun ZG. | Agricultural Equipment & Technology. | 2005; 31: 30-31 |
| 97 | C | [J] | Liu DQ, Chai WB, Gao ZB. | Journal of Anhui Agricultural Sciences. | 2003; 31: 672-673. |
| 98 | C | [J] | Zhao JJ. | Soils and Fertilizers. | 2005; 5: 3-16. |
| 99 | C | [J] | Li JG. | Anhui Agriculture. | 2007; 13: 139, 143. |
| 100 | C | [J] | Sun GY, Gong CH, Wang ZY, Zhu DM, Yuan JH, Zhou CL, Zhang HY, Zhou P. | Anhui Agriculture Science Bulletin. | 2007; 13: 226-118, 134. |
| 101 | C | [M] | Xue YG, Hou LZ, Zhen JQ, Xiong HX, Gao YH, Wang HQ. | Proceedings of Jiangsu Cultivated Land Quality Construction. | 2008. pp. 241-248. |
| 102 | C | [J] | Liu XH | Guizhou Agricultural Sciences. | 2009; 37: 39-41. |
| 103 | C | [J] | Liu GR, Yuan FS, Li ZZ, Liu YR, Luo QX | Acta Agriculturae Jiangxi. | 2001; 13: 1-7. |
| 104 | C | [J] | Wu JF, Zhao XM, Lu ZH, Wang H, Zhu MY, Zhang P. | Acta Agriculturae Universitatis Jiangxiensis. | 2002; 24: 193-195. |
| 105 | C | [M] |  | Annual Report of Major Demonstration Counties of Soil Test and Fertilizer Recommendation Project for Rice in Jiangxi. | 2006-2009 |
| 106 | C | [J] | Huang KY, Dai PA. | Journal of Hunan Agricultural University (Natural Sciences). | 2002; 28: 12-15. |
| 107 | C | [J] | Zou CM, Qin DZ, Gao JS. | Journal of Anhui Technical Teachers College. | 2002; 16: 15-18. |
| 108 | C | [J] | Li DC, Li JM, Xu MG, Qin DZ, Shen HP. | Hunan Agricultural Sciences. | 2004; 3: 23-25, 31. |
| 109 | C | [J] | Li FM, Fan XL, Chen WD. | Plant Nutrition and Fertilizer Science. | 2005; 11: 494-500. |
| 110 | C | [J] | Li MY, Shi QH, Wang T, Pan XH, Tan XM, Zeng YJ. | Acta Agriculturae Universitatis Jiangxiensis. | 2008; 30: 187-193. |
| 111 | C | [J] | Xu C, Wu LH, Zhen ZS, Kong XJ, Ju XT, Zhang FS. | Journal of Zhejiang Agricultural Sciences. | 2003; 2: 75, 76-78. |
| 112 | C | [J] | Nie J, Xiao J, Dai PA, Zheng SX. | Journal of Hunan Agricultural University (Natural Sciences). | 2003; 29: 318-321. |
| 113 | C | [J] | Zhang XM, Guo XS, Li ZF, Song WB, Gui YB, Wang SL, Wang ZS. | Journal of Anhui Agricultural Sciences. | 2006; 34: 4982-4983. |
| 114 | C | [J] | Zhang XM, Guo XS, Li ZF, Wang YL, Liu CS,Hu R. | Chinese Journal of Soil Science. | 2008; 39: 576-581. |
| 115 | C | [J] | Chen ZG, Lu YG, Zhu Q. | Guizhou Agricultural Sciences. | 2006; 34: 50-51. |
| 116 | C | [J] | Yan DZ, Wang DJ, Lin JH. | Acta Pedologica Sinica. | 2005; 42: 440-446. |
| 117 | C | [M] | Gao H, Zhang HC, Dai QG, Zhu DJ, Hu CY. | The Professional Committee of Chinese Crop Cultivation Transition and Academic Seminars. | 2007. pp. 210-215. |
| 118 | C | [J] | Tian ZH, Pan XH. | Journal of Jiangxi Agriculturae Universitatis. | 2007; 29: 894-898. |
| 119 | C | [J] | Hu R, Liu CS. | Anhui Agriculture Science Bulletin. | 2007; 13: 40-42. |
| 120 | C | [J] | Li H, Xu CQ, Li SF, Qian ZH. | Guizhou Agricultural Sciences. | 2008; 36: 39-41. |
| 121 | C | [J] | Wang YQ, Guo XS, Dai MF. | Soil and Fertilizer Sciences in China. | 2008; 2: 31-34. |
| 122 | C | [J] | Pan SG, Cao CG, Cai ML, Wang JP, WangG RH, Yuan BZ, Zhai J, | Plant Nutrition and Fertilizer Science. | 2009; 15: 283-289. |
| 123 | C | [J] | Li SL, Hu C, Qiao Y, Liu GJ, Wan YS, Su ZF. | Hubei Agricultural Sciences. | 2007; 46: 727-729. |
| 124 | C | [J] | Fang KM, Zhong GM, Zhan MS, Wang QS, Gong WL. | Acta Agriculturae Jiangxi. | 2007; 19: 50-53. |
| 125 | C | [J] | Huang X, Xie XL, Xu PZ, Chen JS, Tang SH, Zhang FB, Xie KZ. | Guangdong Agricultural Sciences. | 2008; 5: 47-50, 57. |
| 126 | C | [J] | Cheng SL. | Journal of Hebei Agricultural Sciences. | 2008; 12: 55, 57 |
| 127 | C | [J] | Li YY, Lv HP, Wan XP, Zhou B, Sun MH, Fang HX, Cheng ZQ, | Journal of Anhui Agricultural Sciences. | 2007; 35: 7215-7218. |
| 128 | C | [J] | Xiao HX, Li JG. | Guizhou Agricultural Sciences. | 2007; 35: 80-82. |
| 129 | C | [J] | Lan NR, Zhang XL, Yang CX, Ling HD, Wang LB. | Guizhou Agricultural Sciences. | 2007; 35: 66-68. |
| 130 | C | [J] | Shi QL, Teng SC, Long ZW, Shi JZ, Liu J. | Guizhou Agricultural Sciences. | 2008; 36: 43-45. |
| 131 | C | [J] | Wang HL, He XW. | Soil. | 2009; 41: 320-323. |
| 132 | C | [J] | Zhang GP, Yang MY, Huang ZQ, Wang XQ. | Guizhou Agricultural Sciences. | 2008; 36: 46-47. |
| 133 | C | [J] | He ZM. | Crop Research. | 2002; 16: 26-27. |
| 134 | C | [J] | Zhang QC, Wang GH, Fang B. | Acta Pedologica Sinica. | 2005; 42: 116-121. |
| 135 | C | [J] | Chen J, Li DW, Zhao SQ, Wang C, | China Rice. | 2007; 67-68. |
| 136 | C | [J] | Xiao GB, Liao MQ, Chen GJ, Han RD, Wu ZH. | Acta Agriculturae Jiangxi. | 2007;19: 38-41. |
| 137 | C | [J] | Zhang XM, Guo XS, Li ZF, Xia JF, Hu R, Liu CS, Ye CB. | China Rice. | 2008; 5: 70-72, 73. |
| 138 | C | [J] | Bi CF. | Journal of Anhui Agricultural Science. | 2008; 36: 4473-4474. |
| 139 | C | [J] | Huang ZF | Fujian Agricultural Science and Technology. | 2008; 4: 56, 57. |
| 140 | C | [J] | Liu X, Lin ZF, Wang JE, Zhang XL | Journal of Southern Agriculture. | 2007; 38: 541-543. |
| 141 | C | [J] | Xie ZR, Shen XM, Ye FS, Qian HM, Ji HF, Jing L. | China Rice. | 2006; 2: 40-43. |
| 142 | C | [J] | Wu WG, Ruan XM, Shi FZ. | Journal of Anhui Agricultural Science. | 2007; 35: 1403-1405. |
| 143 | C | [J] | Yang XF, Chen ZX, Zhou Q, Zhang CP, He CP, Li Y, Zhou W. | South China Agriculture. | 2008; 2: 3-5. |
| 144 | C | [J] | Shao YX, Shi JS. | Anhui Agriculture. | 2008; 1: 114, 117. |
| 145 | C | [J] | Su PZ, Liu YP, Zhong YF, Zheng KQ, Zheng XY. | Acta Agriculturae Jiangxi. | 2009; 21: 30-32. |
| 146 | C | [J] | Ma LZ, Quan Z, Chen ZL. | Guangdong Agricultural Sciences. | 2006; 9: 34-35. |
| 147 | C | [J] | Zhang GQ, ChenJn, Li DW, Zhao SQ. | Anhui Agriculture. | 2007; 14: 114. |
| 148 | C | [J] | Guo ZX, Qiu ZA. | Journal of Southern Agriculture. | 2008; 39: 206-209. |
| 149 | C | [J] | Wu ZZ, Mo JH. | Modern Agriculture Science and Technology. | 2009; 6: 133-134. |
| 150 | C | [J] | Wu ZZ, Mo JH. | GanSu Agriculture. | 2008; 6: 93-94, 95. |
| 151 | C | [J] | Yang YH, Qiu YX, Li DX, Huang QY. | Journal of Guangxi Agriculture. | 2008; 23: 5-8. |
| 152 | C | [J] | Gao YG, Yang CL, Li H, Zhou JF, Wang SF, Zhu WH. | Soil and Fertilizer Sciences in China. | 2008; 2: 48-51. |
| 153 | C | [J] | Wang WN, Wang YY, Yao ZQ, Lu MX, Yao SJ, Liu QR, Peng YH, Lu JW. | Hubei Agricultural Sciences. | 2009; 48: 306-309. |
| 154 | C | [J] | Ke XS, Chen J, Li DW, Zhao SQ | Modern Agricultural Sciences and technology. | 2009; 6: 139-143. |
| 155 | C | [J] | Li YJ, Qiu YX, He L, Liu Z. | Modern Agricultural Sciences and technology. | 2009; 2: 121-124. |
| 156 | C | [J] | Wu MF, Wang YZ, Wang WH, Lai YD, Guo KH. | Chinese Agricultural Science Bulletin. | 2008; 24: 280-283. |
| 157 | C | [J] | Lv LW, Wang DZ. | Anhui Agricultural Science Bulletin. | 2008;14: 138-139. |
| 158 | C | [J] | Ye XR, Deng ZL, Zhu CY. | Guangdong Agricultural Sciences. | 2009; 4: 57-59. |
| 159 | C | [J] | Yang CX, Tan J, Du YQ, Cui FH. | Journal of China West Normal University (Natural Sciences). | 2008; 29: 148-152. |
| 160 | C | [J] | Zhang YY. | Anhui Agricultural Science Bulletin. | 2008; 14: 51, 68. |
| 161 | C | [J] | Tian DP, Zhang J. | Modern Agricultural Sciences and technology. | 2008; 15: 49-50, 54. |
| 162 | C | [J] | Wang WN, Wang YY, Yao ZQ, Lu MX, Yao SJ, Liu QR, Peng YH, Lu JW. | Hubei Agricultural Sciences. | 2008; 47: 1416-1419. |
| 163 | C | [J] | Wang LX, Li L, Li JP. | Chinese Agricultural Science Bulletin. | 2009; 25: 155-158. |
| 164 | C | [J] | Wang XQ, Zhang GP, Lu TY. | Guizhou Agricultural Sciences. | 2009; 37: 40-42. |
| 165 | C | [J] | Wang K, BaiZH, Mo GH. | Agricultural Extension Services. | 2009; 26: 63-65. |
| 166 | C | [J] | Wu RC, Bao HC, Wu YQ, Zhang HF. | Agricultural Equipment Technology. | 2009; 35: 38-40. |
| 167 | C | [J] | Wen YN, Wang K. | Guizhou Agricultural Sciences. | 2008; 36: 54-55. |
| 168 | C | [J] | Wang JF, Hu XG, Zhang GC, Liu AP, Zhou DC, Chen Y. | Anhui Agricultural Science Bulletin. | 2009; 15: 96-97. |
| 169 | C | [J] | Li XP, Guo CH, Wang HZ, Chen SZ. | Fujian Science and Technology of Rice and Wheat. | 2005; 23: 15-16. |
| 170 | C | [J] | Zhou JM, Qian XM. | Journal of Zhejiang Agricultural Sciences. | 2007; 4: 436-438. |
| 171 | C | [J] | Long JR, Ma GH, Song CF, Wan YZ, Zhou J, Shen HC. | Research of Agricultural Modernization. | 2008; 29: 112-115, 127. |
| 172 | C | [J] | Wu WG, Yang LS, Su ZS, Zhang YH, Bai YS, Zhao JJ, Hu GS, Fang WJ. | Chinese Journal of Eco-Agriculture. | 2008; 16: 1083-1089. |
| 173 | C | [J] | Zeng YJun, Shi QH, Pan XH, Han T | Acta Agronomica Sinica. | 2008; 34: 1409-1416. |
| 174 | C | [J] | Wang DY, Zhang XF, Shao GS, Xu CM. | Acta Agronomica Sinica. | 2008; 34: 1623-1628. |
| 175 | C | [J] | Dai B, Geng X. | Anhui Agriculture | 2008; 15: 194, 196. |
| 176 | C | [J] | Zhao TJ, Fen YH, Han GG, Dong AL, Pan XS, Song B, Fan WG. | Journal of Anhui Agricultural Sciences. | 2009; 37: 1641-1643 |
| 177 | C | [J] | Zhao TJ, Feng YH, Han GG, Fan WG, Wu ZH, Dong AL, Song B, Pan XS. | Hybrid Rice. | 2009; 24: 57-61. |

*Note*: * C and E denote Chinese and English, respectively; † J, D, M denote Journal (articles), Dissertations, Monographs, respectively.

**Table D.** Frequency distribution, actual area of, and total nitrogen (TN), soil organic carbon (SOC) concentration and pH in different soil grades of inherent productivity for early and late rice in south China and single rice in the Yangtze Delta.

| Rice cropping system | N‡ | Inherent soil productivity†  (kg ha^-1^) | Average yield  (kg ha^-1^) | Frequency distribution  (%) | Area  (10^5^ ha) | TN  (g kg^-1^) | SOC  (g kg^-1^) | Ph |
| --- | --- | --- | --- | --- | --- | --- | --- | --- |
| Early rice | 185 | <3500 | 3103 (±310)* | 21.5 | 12.5 | 1.4 (±0.6) | 16.87 (±6.18) | 5.73 (±0.86) |
|  | 498 | 3500-5000 | 4379 (±403) | 57.8 | 33.5 | 1.4 (±0.4) | 18.87 (±8.68) | 5.75 (±0.90) |
|  | 178 | >5000 | 5192 (±108) | 20.7 | 12.0 | 1.5 (±0.5) | 19.89 (±8.24 ) | 5.66 (±0.83) |
| Late rice | 225 | <4000 | 3522 (±513) | 21.3 | 13.3 | 1.2 (±0.4) | 17.45 (±7.13 ) | 5.95 (±0.80) |
|  | 692 | 4000-5500 | 4807 (±416) | 65.6 | 40.8 | 1.4 (±0.6) | 18.06 (±7.55) | 5.77 (±0.81) |
|  | 138 | >5500 | 5640 (±84) | 13.1 | 8.2 | 1.5 (±0.5) | 18.83 (±5.50) | 5.53 (±0.84) |
| Single rice | 574 | <4500 | 3932 (±461) | 16.7 | 20.9 | 1.3 (±0.5) | 13.39 (±6.95) | 6.21 (±0.85) |
|  | 1199 | 4500-6000 | 5346 (±405) | 34.9 | 43.7 | 1.5 (±1.3) | 14.07 (±6.57) | 6.28 (±1.01) |
|  | 1419 | 6000-7500 | 6632 (±407) | 41.3 | 51.7 | 1.6 (±1.1) | 15.25 (±6.06) | 6.29 (±0.97) |
|  | 244 | >7500 | 7720 (±140) | 7.1 | 8.9 | 1.6 (±0.6) | 16.18 (±6.43) | 6.06 (±1.01) |

*Note*: ‡Yield data of zero-N plots were collected from 5351 on-farm trials conducted for 1-2 years during 2000-2012; N represent data size for each grade of rice farming types. †Inherent soil productivity was estimated as yields in zero-N plots and graded based on classification systems of arable land quality in China [5] *Average yield of all data for each of grade..The literature sources and documents from which the data were derived are listed in Table C.

**References**

1. Dobermann A, Witt C, Abdulrachman S, Gines HC, Nagarajan R, Son TT, et al. Estimating indigenous nutrient supplies for site-specific nutrient management in irrigated rice. Agron J. 2003; 95(4): 924-935.

2. Cassman KG, Gines GC, Dizon MA, Samson MI, Alcantara JM. Nitrogen-use efficiency in tropical lowland rice systems: contributions from indigenous and applied nitrogen. Field Crop Res. 1996; 47(1): 1-12.

3. Dobermann A, Witt C, Dawe D, Abdulrachman S, Gines GC, Nagarajan R, et al. Site-specific nutrient management for intensive rice cropping systems in Asia. Field Crop Res. 2002; 74(1): 37-66.

4. Fan MS, Lal R, Cao J, Qiao L, Jiang RF, Zhang FS. Plant-based assessment of inherent soil productivity and contributions to China’s cereal crop yield increase since 1980. PLoS ONE. 2013; 8(9): e74617.

5. Survey and Classification of China’s Cultivated Land Quality. Beijing: Ministry of Land and Resources of China; 2009 (in Chinese).

6. China Agriculture Statistical Report. Beijing: Ministry of Agriculture of China, China Agriculture Press; 2011 (in Chinese).

7. Zhang FS, Cui ZL, Chen XP, Ju XT, Shen JB, Chen Q, et al. Integrated nutrient management for food security and environmental quality in China. Advan Agron. 2012; 116: 1-32.

8. Zhang FS, Chen XP Cui ZL,Chen Q, Fan MS, et al. Technological guides for high yield and high nutrient use efficency of major crops, China Agricultural University press, Beijing, 2011.

9. Zhang H, Chen TT, Wang ZQ, Yang JC, Zhang JH. Involvement of cytokinins in the grain filling of rice under alternate wetting and drying irrigation. J Exp Bot. 2010; 61(13): 3719-3733.

10. Yan XY, Yagi K, Akiyama H, Akimoto H. Statistical analysis of the major variables controlling methane emission from rice fields. Global Change Biol. 2005; 11(7): 1131-1141.

11. Maclean JL, Daw [DC,](http://book.douban.com/search/D C Daw) Hardy B, Hettel [GP.](http://book.douban.com/search/G P Hettel) Rice almanac. Wallingford: CABI Publishing; 2002.

12. Forster P, Ramaswamy V, Artaxo P,  Berntsen T, Betts R,  Fahey DW, et al. Changes in atmospheric constituents and in radiative forcing. In: Solomon S, Qin D, Manning M, Chen Z, Marquis M, editors. Climate change 2007: the physical science basis. Contribution of working group I to the fourth assessment report of the intergovernmental panel on climate change. Cambridge: Cambridge University Press; 2007. pp. 129-234.

13. Mosier AR, Halvorson AD, Reule CA, Liu X. Net global warming potential and green house gas intensity in irrigated cropping systems in northeastern Colorado. J Environ Qual. 2006; 35: 1584-1598.

14. Searchinger T. Heimlich R, Houghton RA, Dong FX, Elobeid A, Fabiosa J, et al. Use of U.S. croplands for biofuels increases greenhouse gases through emissions from land-use change. Science. 2008; 319: 1238-1240.

15. Burney JA, Davis SJ, Lobell DB. Greenhouse gas mitigation by agricultural intensiﬁcation. Proc Natl Acad Sci USA. 2010; 107(26): 12052-12057.

16. The National Land Control Planning (2011-2015). Beijing: Ministry of Land and Resources of China; 2012 (in Chinese).
