# Supplementary material for: Exploiting Co-Benefits of Increased Rice Production and Reduced Greenhouse Gas Emission through Optimized Crop and Soil Management
Source: PLoS One. 2015 Oct 9;10(10):e0140023. doi: 10.1371/journal.pone.0140023 (PMC4599856; doi:10.1371/journal.pone.0140023)
Supplement: S2 Text — (DOC) [file pone.0140023.s006.doc]

**Supporting information for**

**Exploiting co-benefits of increased rice production and reduced greenhouse gas emission through optimized crop and soil management**

Ning An^1^, Mingsheng Fan^1*^, et al.,

*To whom correspondence should be addressed. Email: [fanms@cau.edu.cn](mailto:fanms@cau.edu.cn), Tele: 86-10-62731661; Fax: 86-10-62731016

**S2Text Supplementary discussions**

**1. Comparison of N_2_O emission factors and CH_4_ emissions between the current and previous studies**

N_2_O emissions of N fertilization are often expressed as a function of rates of fertilizer N application. Earlier studies adopted a linear relationship to estimate N_2_O emissions based on the amount of N added to agricultural soils [1]. However, some recent studies have reported exponential increases in direct N_2_O emissions with increasing N application rates, indicating that a larger proportion of N is lost at high N application rates or exceeding crop N uptake [2-5].

to those 10.,r time horizon oil d.uction is evidence. and 000000000000000000000000000000000000000000000000000000000000000000000

his ? model than linear one. Tals. fit to account for the relationship between 0000000000000000000000000000000000000000000000The results of our meta-analysis indicate that linear and exponential models of the fertilizer N application rate to N_2_O emissions during the rice growing season were best fit curves for F-D-F (FPs) and F-D-F-M (BMPs), respectively (S2 Fig.).

The fertilizer-induced emission factor for N_2_O during the rice growing season for F-D-F was 0.33 % with a standard error of 0.08%. Seasonal background N_2_O emission was estimated to be 0.23 kg N_2_O-N ha^-1^ with a standard error (0.16 kg N_2_O-N ha^-1^). Using literature-based statistical models [6], it was showed that the country-specific emission factors of N_2_O for paddy soils were 0.42% with a standard error 0.06% for F-D-F. Seasonal background N_2_O emission was negligible under the water regime of F-D-F. In view of the standard errors in the two studies, emission factors and background N_2_O emission of the previous [6] and in the current study were broadly comparable. The differences in establishing the statistical model of fertilizer-induced direct N_2_O emissions between the previous [6] and the current study were: (1) the references involved in assessment for the latter were on double rice in south China and single rice in the Yangtze Delta, while the former on whole rice production systems included those in north, north east and south China and in the Yangtze Delta; (2) we have not included treatments with organic amendments such as manures and crop residues for assessments as did the previous [6]; (3) published data on N_2_O emissions after 2005, which are omitted in the previous study [6], were included in the current study. Thus, the emission factor 0.0033 kg N_2_O-N kg N ha^-1^ was adopted to estimate the fertilizer-induced direct N_2_O emissions from rice paddies under the water regime of F-D-F.

The nonlinear models between direct N_2_O emissions and increasing N application rates or N surplus for paddy soil also exist [3,5,7]. However, these studies either focus on specific rice production domain and/or do not distinguish the effects of water management (e.g. F-D-F vs F-D-F-M) on N_2_O emissions. We therefore adopted the current established exponential model to estimate N_2_O emissions during the rice growing season for F-D-F-M for 403 on-farm trials (S2 Fig.). F-D-F-M led to larger N_2_O emissions than traditional practices of F-D-F because of the alternating dry and wet conditions after midterm drainage creating a favorable soil environment for both nitrification and denitrification processes which contributed greatly to higher N_2_O emissions under the F-D-F-M water regime [8].

CH_4_ emission in paddy soil showed regional and rice farming type specific features [9,10,11]. With the intent to check the interactive effects of inherent soil productivity and management factors at rice farming type scale on agronomic productivity and environmental footprint, we thus used previous developing statistical model [9] to evaluate CH_4_ emission based on soil inherent productivity grades and rice farming types. This model relates CH_4_ ﬂux in the rice growing season to SOC and soil pH, water regime in the rice growing season, water status in the previous season, organic amendment and climate, and has been suggested for assessment of national or regional CH_4_ emission inventories.

In terms of rice farming types (single rice refer to rice under rice-upland rotation in the Yangtze River Basin, while early and late refer to rice under double rice system of south of China in this study), the average CH_4_ emissions across soil inherent productivity grades and management practices in the current study followed the order of late > early > single rice, which were 129 (115-143) kg ha^-1^ for single rice, 213 (190-235) kg ha^-1^ for early rice and 504 (457-548) kg ha^-1^ for late rice systems. The similar results could be found in direct measurements in the field condition for single rice of Yangtze River Basin and double rice systems of south of China [11-13]. The differences in CH_4_ emissions between the types of rice cropping systems may be due to pre-season water regime, SOC concentration and climate [9,14,15]. BMPs led to decrease CH_4_ emission on average by 14.3 % than FPs, this was mainly due to shift in water management from F-D-F in FPs to F-D-F-M in BMPs. A previous study which integrated analyzed 67 field experimental results about CH_4_ emissions (13 of F-D-F Vs 54 of F-D-F-M) from rice field in China showed that F-D-F-M could lead to decrease CH_4_ flux than F-D-F regimes, but with great variations (5.21±0.66 mg m^-2^ h^-1^ for former and 3.8±1.11 mg m^-2^ h^-1^ for latter) [10].

**2. Reliability and limitations of the current study**

Rice yield and production data of FPs in the current study were very similar to national statistical data [16]. This may be attributed to on-farm trials having been conducted in soils with different inherent productivities over major double and single rice systems. The attainable yields were estimates of landscape-scale achievable yield during 2008-2011 and not estimates of physiologically achievable potential yields. Thus, yield gap being the difference between FP yields on soils with diverse productivity levels represents the practical and exploitable yield gap. Furthermore, soil and management practices (FPs vs BMPs) and rice farming types were taken into consideration for integrated assessment. Thus, the current study may quite accurately reflect options for increasing rice productivity and production and adding more value to the system with a smaller environmental footprint by following various strategies such as increasing soil inherent productivity and/or adoption of better crop, nutrient and water management in the near and medium term.

Some uncertainties of the current study may be derived from the plant-based approach in assessment of inherent soil productivity. A significant and strong regression in yield between zero-N and fertilizer-omission plots (S3 Fig.) confirms that N deficiency was a general and key limiting factor in irrigated rice systems [17,18]; and shows that yield data from zero-N or fertilizer-omission plots may mainly reflect the interacting soil properties and serve as indicator of inherent soil productivity for major Chinese paddy soils. However, the regression equations accounted for about 77 % of the variation for the three rice farming systems (S3 Fig.). This implies that indicators such as yield data in zero-N plots may underestimate the constraints of other soil nutrient supply capacities and associated soil processes to inherent soil productivity. It is likely that the total area of low productivity soils may have been underestimated. Further, with the intent to check the interactive effects of biophysical factors (such as soil and rice farming type) versus management factors on agronomic productivity and environmental footprint, we checked the performance management practices at various soil inherent productivity levels at rice type scale. The latter represents agroecological zones (AEZs) on the basis of temperature and rainfall (e.g. single rice in warm sub-humid subtropics and double rice in warm/cool humid subtropics), and seasonal climate characters within the warm/cool humid subtropics (early rice vs late rice). However, in view of variations in climatic variables such as temperature, sunshine and rainfall among specific locations and experimental years even within same rice farming type, uncertainty in current study still persists. Where possible, those factors should be taken into account in assessment.

Another source of uncertainty may come from assessment in GHG emissions. Though, water management is key factor in determining differences in N_2_O emission between FPs and BMPs. The time of N fertilizer application is quiet similar between FPs and BMPs. It is quiet possible that with the right fine-tuning of N and crop management under BMPs N_2_O emissions can be kept smaller than estimate in current study [19]. We assessed CH_4_ emission at rice farming types scale to explore the differences between FPs and BMPs upon adopted on various soil inherent productivity grades. The results reflected the effects of major factors such as rice farming types, soil inherent productivity grades (referred as SOC and soil pH) and water management regimes in both rice growing seasons (F-D-F and F-D-F-M) and pre-season on CH_4_ emission. However, CH_4_ emission can also be affected by other soil-related factors such as soil texture, soil thermal conductivity and total N [11,20-23], and plant related to factors e.g. rice cultivar [24-27] and aboveground biomass [28]. The further efforts should be taken to produce more precise pictures by direct field measurements on multiple-locations if possible. A detailed, process-based model such as DNDC also help to do a good job for up-scaling site-specific simulation of CH_4_ to region with many specific observations, such as microbial activity, soil thermal conductivity and hydraulic conductivity [29,30].

**Reference**

1. Eggleston S, Buendia L, Miwa K, Ngara T, Tanabe K. 2006 IPCC guidelines for national greenhouse gas inventories, volume 2: energy. Japan: Institute for Global Environmental Strategies; 2006.

2. Hoben JP, Gehl RJ, Millar N, Grace PR, Robertson GP. Nonlinear nitrous oxide (N_2_O) response to nitrogen fertilizer in on-farm corn crops of the US Midwest. Global Change Biol. 2011; 17(2): 1140-1152.

3. Xia YQ, Yan XY. Ecologically optimal nitrogen application rates for rice cropping in the Taihu Lake region of China. Sustain Sci. 2012; 7(1): 33-44.

4. Pittelkow CM, Adviento-Borbe MA, Hill JE, Six J, Kessel CV, Linquist BA. Yield-scale global warming potential of annual nitrous oxide and methane emissions from continuously flooded rice in response to nitrogen input. Agric Ecosyst Environ. 2013; 177: 10-20.

5. Pittelkow CM, Adviento-Borbe MA, Kessel CV, Hill JE, Linquist BA. Optimizing rice yields while minimizing yield-scaled global warming potential. Global Change Biol. 2014; 20(5): 1382-1393.

6. Zou JW, Huang Y, Qin YM, Liu SW, Shen QR, Pan GX, et al. Changes in fertilizer-induced direct N_2_O emissions from paddy fields during rice-growing season in China between 1950s and 1990s. Global Change Biol. 2009; 15(1): 229-242.

7. Cui ZL, Wang GL, Yue SC, Wu L, Zhang WF, Zhang FS, et al. Closing the N-use

efficiency gap to achieve food and environmental security. Environ Sci Technol.

2014; 48: 5780-5787.

8. Dobbie KE, Smith KA. Nitrous oxide emission factors for agricultural soils in Great Britain: impacts of soil water-filled pore space and other controlling variables. Global Change Biol. 2003; 9: 204-218.

9. Yan XY, Yagi K, Akiyama H, Akimoto H. Statistical analysis of the major variables controlling methane emission from rice fields. Global Change Biol. 2005; 11(7): 1131-1141.

10. Shi SW, Li YE, Liu YT, Wan YF, Gao QZ, Zhang ZX. CH_4_ and N_2_O emission from rice field and mitigation options based on field measurements in China: an integration analysis. Sci Agric Sin. 2010; 43(14) (in Chinese with English abstract).

11. Wei HP, Sun WJ, Huang Y. Statistical analysis of methane emission from rice fields in China and the driving factors. Sci Agric Sin. 2012; 45(17): 3531-3540 (in Chinese with English abstract).

12. Shang QY, Yang XX, Gao CM, Wu PP, Liu JJ, Xu YC, et al. Net annual global warming potential and greenhouse gas intensity in Chinese double rice-cropping systems: a 3-year field measurement in long-term fertilizer experiments. Global Change Biol. 2011; 17(6): 2196-2210.

13. Ma YC, Kong XW, Yang B, Zhang XL, Yan XY, Yang JC, et al. Net global warming potential and greenhouse gas intensity of annual rice-wheat rotations with integrated soil-crop system management. Agric Ecosyst Environ. 2013; 164: 209-219.

14. Holzapfel-Pschorn A, Seiler W. Methane emission during a cultivation period from an Italian rice paddy. J Geophys Res. 1986; 91(D11): 11803-11814.

15. Neue HU, Roger PA. Potential of methane emission in major rice ecologies. In: Zepp RG. editor. Climate biosphere interaction: biogenic emissions and environmental effects of climate change. New York: John Wiley and Sons; 1994. pp. 65-93.

16. China Statistical Yearbook. Beijing: National Bureau of statistics of China, China Statistics Press. 2011; Available: http://data. stats.gov.cn/publish.

17. Dobermann A, Witt C, Dawe D, Abdulrachman S, Gines GC, Nagarajan R, et al. Site-specific nutrient management for intensive rice cropping systems in Asia. Field Crop Res. 2002; 74(1): 37-66.

18. Dobermann A, Witt C, Abdulrachman S, Gines HC, Nagarajan R, Son TT, et al. Estimating indigenous nutrient supplies for site-specific nutrient management in irrigated rice. Agron J. 2003; 95(4): 924-935.

19. Adviento-Borbe MAA, Haddix ML, Binder DL, Walters DT, 0000000000000000000000000000000000000000000000000000000000000000000000000000000000000000000000000000000000000000000000000000Dobermann A. Soil greenhouse gas fluxes and global warming potential in four high-yielding maize systems. Global Change Biol. 2007; 13: 1972-1988.

20. Bachelet D, Neue HU. Methane emission from wetland rice areas of Asia. Chemosphere. 1993; 26(1-4): 219-237.

21. Neue HU. Methane emission from rice fields. Bioscience. 1993; 43(7): 466-474.

22. Achtnich C, Bak F, Conrad R. Competition for electron donors among nitrate reducers, ferric iron reducers, sulfate reducers, and methanogens in anoxic paddy soil. Biol Fert Soils. 1995; 19(1): 65-72.

23. Cai ZC, Xing GX, Shen GY, Xu H, Yan XY, Tsuruta H, et al. Measurements of CH_4_ and N_2_O emissions from rice paddies in Fengqiu China. Soil Sci Plant Nutr. 1999; 45: 1-13.

24. Wang B, Xu Y, Wang Z, Li Z, Guo Y, Shao K, et al. Methane emissions from rice fields as affected by organic amendment, water regime, crop establishment, and rice cultivar. Environ Monit Assess. 1999; 57(2): 213-228.

25. Jain MC, Kumar S, Wassmann R, Mitra S, Singh SD, Singh JP, et al. Methane emissions from irrigated rice fields in Northern India (New Delhi). Nutr Cycl Agroecosys. 2000; 58(1-3): 75-83.

26. Wassmann R, Buendia LV, Lantin RS, Bueno CS, Lubigan LA, Umali A, et al. Mechanisms of crop management impact on methane emissions from rice fields in Los Baños, Philippines. Nutr Cycl Agroecosys. 2000; 58: 107-119.

27. Aulakh MS, Wassmann R, Rennegberg H. Methane emissions from rice field- quantification, mechanisms, role of management, and mitigation options. Adv Agron. 2001; 70: 193-260.

28. Sass RL, Fisher FM, Harcombe PA. Methane production and emission in a Texas rice field. Global Biogeochem Cycles. 1990; 4(1): 47-68.

29. Li CS, Mosier A, Wassmann R, Wassmann R, Cai ZC, Zheng XH, et al. Modeling greenhouse gas emissions from rice-based production systems: sensitivity and upscaling. Global Biogeochem Cycles. 2004; 18(1): GB1043, doi:10.1029/2003GB002045.

30. Kraus D, Weller S, Klatt S, Haas E, Wassmann R, Kiese R, et al. A new Landscape DNDC biogeochemical module to predict CH_4_ and N_2_O emissions from lowland rice and upland cropping systems. Plant Soil. 2015; 386(1-2): 125-149.
